# Diagnosis and treatment of Hymenoptera venom allergy: S2k Guideline of the German Society of Allergology and Clinical Immunology (DGAKI) in collaboration with the Arbeitsgemeinschaft für Berufs- und Umweltdermatologie e.V. (ABD), the Medical Association of German Allergologists (AeDA), the German Society of Dermatology (DDG), the German Society of Oto-Rhino-Laryngology, Head and Neck Surgery (DGHNOKC), the German Society of Pediatrics and Adolescent Medicine (DGKJ), the Society for Pediatric Allergy and Environmental Medicine (GPA), German Respiratory Society (DGP), and the Austrian Society for Allergy and Immunology (ÖGAI)

**DOI:** 10.5414/ALX02430E

**Published:** 2023-10-02

**Authors:** Franziska Ruëff, Andrea Bauer, Sven Becker, Randolf Brehler, Knut Brockow, Adam M. Chaker, Ulf Darsow, Jörg Fischer, Thomas Fuchs, Michael Gerstlauer, Sunhild Gernert, Eckard Hamelmann, Wolfram Hötzenecker, Ludger Klimek, Lars Lange, Hans Merk, Norbert K. Mülleneisen, Irena Neustädter, Wolfgang Pfützner, Wolfgang Sieber, Helmut Sitter, Christoph Skudlik, Regina Treudler, Bettina Wedi, Stefan Wöhrl, Margitta Worm, Thilo Jakob

**Affiliations:** 1 Department of Dermatology and Allergy, LMU University Hospital, Munich,; 2 Department of Dermatology, University Hospital Carl Gustav Carus, Technical University Dresden, Dresden,; 3 Department of Otorhinolaryngology, Head and Neck Surgery, University of Tuebingen, Tübingen,; 4 Department of Dermatology, Münster University Hospital, Münster,; 5 Department of Dermatology and Allergology Biederstein, Faculty of Medicine, Technical University of Munich, Munich,; 6 Department of Otorhinolaryngology Klinikum rechts der Isar, Faculty of Medicine, Technical University of Munich, Munich,; 7 University Hospital for Dermatology and Allergology, Clinic Oldenburg, Oldenburg,; 8 Department of Dermatology, Venereology and Allergology, University Medical Center Göttingen, Göttingen,; 9 Clinic for Children and Adolescents, University Hospital Augsburg, Augsburg,; 10 Pediatric Clinic, Marienhospital Bonn, GFO Kliniken, Bonn,; 11 Children’s Center Bethel, University Hospital OWL, Bielefeld University, Bielefeld, Germany,; 12 Department of Dermatology, Kepler University Hospital, Medical Faculty of University Linz, Linz, Austria,; 13 Center for Rhinology and Allergology, Wiesbaden,; 14 Department of Dermatology and Allergology, University Hospital of RWTH Aachen University, Aachen,; 15 Center for Asthma and Allergy, Leverkusen,; 16 Cnopfsche Paediatric Clinic, Nuremberg,; 17 Department of Dermatology and Allergology, University Hospital Marburg, Philipps-Universität Marburg, Marburg,; 18 Hospital Wörth an der Donau, Wörth an der Donau,; 19 Institute for Theoretical Surgery, Philipps-University Marburg, Marburg,; 20 Institute for Interdisciplinary Dermatological Prevention and Rehabilitation (iDerm) at the University of Osnabrueck, Osnabrueck, and BG Clinic Hamburg, Hamburg,; 21 University Leipzig Medical Faculty, Leipzig,; 22 Comprehensive Allergy, Department of Dermatology and Allergy, Hannover Medical School, Hanover, Germany,; 23 Floridsdorf Allergy Center (FAZ), Vienna, Austria,; 24 Department of Dermatology, Venereology and Allergology, Charité-Universitätsmedizin Berlin, Campus Charité Mitte, Berlin, and; 25 Department of Dermatology and Allergology, University Hospital Giessen, Justus Liebig University Gießen, Gießen, Germany

**Keywords:** allergy, anaphylaxis, bee venom, diagnostics, Hymenoptera venom, insect sting reaction, emergency therapy, venom immunotherapy, Vespula venom

## Abstract

Hymenoptera venom (HV) is injected into the skin during a sting by Hymenoptera such as bees or wasps. Some components of HV are potential allergens and can cause large local and/or systemic allergic reactions (SAR) in sensitized individuals. During their lifetime, ~ 3% of the general population will develop SAR following a Hymenoptera sting. This guideline presents the diagnostic and therapeutic approach to SAR following Hymenoptera stings. Symptomatic therapy is usually required after a severe local reaction, but specific diagnosis or allergen immunotherapy (AIT) with HV (VIT) is not necessary. When taking a patient’s medical history after SAR, clinicians should discuss possible risk factors for more frequent stings and more severe anaphylactic reactions. The most important risk factors for more severe SAR are mast cell disease and, especially in children, uncontrolled asthma. Therefore, if the SAR extends beyond the skin (according to the Ring and Messmer classification: grade > I), the baseline serum tryptase concentration shall be measured and the skin shall be examined for possible mastocytosis. The medical history should also include questions specific to asthma symptoms. To demonstrate sensitization to HV, allergists shall determine concentrations of specific IgE antibodies (sIgE) to bee and/or vespid venoms, their constituents and other venoms as appropriate. If the results are negative less than 2 weeks after the sting, the tests shall be repeated (at least 4 – 6 weeks after the sting). If only sIgE to the total venom extracts have been determined, if there is double sensitization, or if the results are implausible, allergists shall determine sIgE to the different venom components. Skin testing may be omitted if in-vitro methods have provided a definitive diagnosis. If neither laboratory diagnosis nor skin testing has led to conclusive results, additional cellular testing can be performed. Therapy for HV allergy includes prophylaxis of reexposure, patient self treatment measures (including use of rescue medication) in the event of re-stings, and VIT. Following a grade I SAR and in the absence of other risk factors for repeated sting exposure or more severe anaphylaxis, it is not necessary to prescribe an adrenaline auto-injector (AAI) or to administer VIT. Under certain conditions, VIT can be administered even in the presence of previous grade I anaphylaxis, e.g., if there are additional risk factors or if quality of life would be reduced without VIT. Physicians should be aware of the contraindications to VIT, although they can be overridden in justified individual cases after weighing benefits and risks. The use of β-blockers and ACE inhibitors is not a contraindication to VIT. Patients should be informed about possible interactions. For VIT, the venom extract shall be used that, according to the patient’s history and the results of the allergy diagnostics, was the trigger of the disease. If, in the case of double sensitization and an unclear history regarding the trigger, it is not possible to determine the culprit venom even with additional diagnostic procedures, VIT shall be performed with both venom extracts. The standard maintenance dose of VIT is 100 µg HV. In adult patients with bee venom allergy and an increased risk of sting exposure or particularly severe anaphylaxis, a maintenance dose of 200 µg can be considered from the start of VIT. Administration of a non-sedating H1-blocking antihistamine can be considered to reduce side effects. The maintenance dose should be given at 4-weekly intervals during the first year and, following the manufacturer’s instructions, every 5 – 6 weeks from the second year, depending on the preparation used; if a depot preparation is used, the interval can be extended to 8 weeks from the third year onwards. If significant recurrent systemic reactions occur during VIT, clinicians shall identify and as possible eliminate co-factors that promote these reactions. If this is not possible or if there are no such co-factors, if prophylactic administration of an H1-blocking antihistamine is not effective, and if a higher dose of VIT has not led to tolerability of VIT, physicians should should consider additional treatment with an anti IgE antibody such as omalizumab as off lable use. For practical reasons, only a small number of patients are able to undergo sting challenge tests to check the success of the therapy, which requires in-hospital monitoring and emergency standby. To perform such a provocation test, patients must have tolerated VIT at the planned maintenance dose. In the event of treatment failure while on treatment with an ACE inhibitor, physicians should consider discontinuing the ACE inhibitor. In the absence of tolerance induction, physicians shall increase the maintenance dose (200 µg to a maximum of 400 µg in adults, maximum of 200 µg HV in children). If increasing the maintenance dose does not provide adequate protection and there are risk factors for a severe anaphylactic reaction, physicians should consider a co-medication based on an anti-IgE antibody (omalizumab; off-label use) during the insect flight season. In patients without specific risk factors, VIT can be discontinued after 3 – 5 years if maintenance therapy has been tolerated without recurrent anaphylactic events. Prolonged or permanent VIT can be considered in patients with mastocytosis, a history of cardiovascular or respiratory arrest due to Hymenoptera sting (severity grade IV), or other specific constellations associated with an increased individual risk of recurrent and/or severe SAR (e.g., hereditary α-tryptasemia). In cases of strongly increased, unavoidable insect exposure, adults may receive VIT until the end of intense contact. The prescription of an AAI can be omitted in patients with a history of SAR grade I and II when the maintenance dose of VIT has been reached and tolerated, provided that there are no additional risk factors. The same holds true once the VIT has been terminated after the regular treatment period. Patients with a history of SAR grade ≥ III reaction, or grade II reaction combined with additional factors that increase the risk of non response or repeated severe sting reactions, should carry an emergency kit, including an AAI, during VIT and after regular termination of the VIT.

## 1. Preliminary remarks 

### 1.1. Objective and development of the guideline 

The aim of this guideline is to summarize the current state of knowledge on the diagnosis and treatment of honeybee and vespid venom allergy and to provide recommendations for clinical practice. Large local reactions, intoxications, or reactions to stings of other insects are mentioned only for differential diagnosis. This guideline is intended for physicians who provide allergological care to patients with honeybee or vespid venom allergy. 

This guideline updates the S2k guideline on Hymenoptera venom allergy (HVA) published in 2011 [1]. It takes into account the methodological guidelines of the Association of the Scientific Medical Societies in Germany (AWMF) for the development of guidelines for diagnosis and therapy and follows the three-step concept of the AWMF for the development of a S2k guideline (https://www.awmf.org/regelwerk/regeln-fuer-das-ll-register). The DELBI criteria are considered [2]. Further details of the methodology can be found in the guideline report on the AWMF website (https://www.awmf.org/leitlinien/). 

This guideline is based on a systematic review by the European Academy of Allergy and Clinical Immunology (EAACI) in 2018 of the previously published literature [3], as well as relevant trials and meta-analyses published since then. These studies were identified through a systematic literature search of PubMed and with specific reference to the current EAACI guideline on the diagnosis and treatment of HVA [4]. The consensus for this guideline was reached independently of the European guideline by the committee listed in the authors‘ list.[Table Abbreviations]

### 1.2. Participating professional societies and consensus building 

The guideline was developed under the leadership of members of the Working Group on Insect Venom Allergy of the German Society for Allergology and Clinical Immunology (DGAKI). The participating medical societies and their representatives are listed in Table 1. Funding was provided by the DGAKI. 

The consensus process was as follows: In October 2019, representatives for the expert group were nominated by the scientific societies. In October 2021, a draft guideline was made available to the commission members. The revised draft, taking into account the written comments of the Commission members, was discussed and approved in virtual consensus conferences on November 2, 2021, June 9, 2022, and July 7, 2022. These consensus conferences were moderated and scientifically accompanied by the external neutral moderator Priv.-Doz. Dr. Helmut Sitter. Subsequently, the draft was submitted to all relevant bodies of the participating societies for approval and recommendation for adoption. The final approval was formally completed by August 2, 2023. 

### 1.3. Recommendations and consensus 

The strength of consensus indicated in the recommendations was defined as follows: strong consensus > 95%, consensus > 75 – 95%, majority agreement > 50 – 75%, disagreement < 50%. 

The strength of each recommendation is expressed in this guideline using standardized terms (Table 2). In the manuscript, a strong recommendation is indicated by “we recommend” or “shall” and a conditional or weakened recommendation by “we suggest” or “should”. An open recommendation is indicated by “can”. 

## 2. Triggers, clinic, and epidemiology of exaggerated sting reactions 

### 2.1. Hymenoptera 

The Hymenoptera comprise more than 100,000 known species of insects worldwide. A subgroup of Hymenoptera are the stinging insects (Aculeata), of which the females have a venomous sting that injects venom into the human skin when stinging. Some components of Hymenoptera venom are potential allergens and can cause large local and systemic allergic reactions (SAR) after IgE-mediated sensitization. 

In central Europe, honeybees (*Apis mellifera*; hereafter referred to as bees) and certain vespids (especially *Vespula vulgaris*, *V. germanica*; hereafter referred to as vespids) are the most common elicitors of clinically significant sting reactions. Less frequently, other Hymenoptera such as yellowjackets (*Dolichovespula* spp.), paper wasps (*Polistes* spp.), hornets (*Vespa* spp.) or bumblebees (*Bombus* spp.) are responsible for sting reactions in Central Europe, and only sporadically stings are caused by native ants (*Formicidae* spp.). Fire ants (*Solenopsis invicta*) have also been implicated in anaphylactic sting reactions in other continents. They have not yet established stable local populations in Europe. However, changes in climatic conditions in Europe could lead to the emergence or spread of previously non-native insect species. 

Hematophagous insects continuously secrete saliva during sucking. This saliva contains biogenic amines, vasoactive peptides, anticoagulant proteins, and digestive enzymes. These substances can also induce allergic reactions, and therefore stings by hematophagous insects should be considered in the differential diagnosis as very rare triggers of systemic allergic sting reactions. 

### 2.2. Clinical symptoms of sting reactions 

**2.2.1. Local reactions and non-specific general reactions**

The venom administered into the skin causes an undesirable but normal local reaction with usually immediate pain followed by redness, swelling, and itching. A normal local reaction is said to have occurred if the swelling, usually erythematous, at the site of the sting is < 10 cm in diameter and tends to subside within 24 hours. 

A sting in the respiratory tract can cause obstruction and be life-threatening even if the swelling is only local. 

The term “large local reaction” is used when the diameter of the swelling is > 10 cm and persists for more than 24 hours [5]. In ~ 80% of large local reactions, the diameter of the swelling is between 10 and 20 cm, and in ~ 20% it is more than 20 cm [6]. On average, large local reactions subside after ~ 7 days (range 1 – 21 days) [6]. Stings near joints and stings in parts of the body with good blood circulation (e.g., the face) may cause large local reactions, which should not be confused with SARs, as there are no specific allergic immediate-type symptoms. Especially in children, a non-infectious systemic inflammatory reaction with or without general symptoms such as chills, malaise, or shivering and/or a non-infectious lymphangitis may occur, typically on the first or second day after the sting. In unclear clinical situations, the determination of inflammatory parameters (e.g., procalcitonin, differential blood count) may be helpful in the differential diagnosis of a bacterial skin/soft tissue infection. 

**2.2.2. Systemic reactions**

2.2.2.1. Systemic allergic reaction (anaphylaxis)

A systemic allergic reaction (anaphylaxis) is the most common form of hypersensitivity to Hymenoptera stings and is usually caused by a single sting. The reaction is triggered by HV-specific IgE antibodies (HV sIgE) directed against venom components. Cross-linking of these mast cell-bound antibodies by allergens leads to the initiation of an intracellular signaling cascade culminating in the release of symptom-triggering mediators (e.g., histamine). 

Symptoms include generalized skin reactions (flushing, urticaria, angioedema), mild to moderate respiratory, cardiovascular, or gastrointestinal symptoms, and severe symptoms including severe airway obstruction, anaphylactic shock (often with loss of consciousness) and respiratory/circulatory arrest (Table 3). According to Ring and Messmer [7], systemic reactions of grade I are classified as mild, those of grade II as moderate, those of grade III as severe and those of grade IV as very severe, requiring resuscitation. In severe and very severe anaphylaxis in adults, symptoms of skin involvement may be completely absent [8, 9, 10], leaving cardiovascular failure as the main symptom. In this acute situation, it may be difficult to distinguish anaphylaxis from reactions caused primarily by cardiovascular disease. 

When anaphylaxis results in death, male adults are more likely to die [11] and death is usually due to cardiovascular failure [11, 12, 13]. Fatal outcome of an anaphylactic reaction in children is very rare; in such cases, the fatal outcome is usually caused by symptoms developing in the lower respiratory tract [12]. 

2.2.2.2. Intoxication

Toxin exposure can cause severe disease patterns with symptoms such as rhabdomyolysis and hemolysis and sequential organ damage if the number of stings is high [5, 14]. Young children are particularly vulnerable [15, 16]. However, hundreds of stings can be survived without harm with timely symptomatic intervention [17]. 

2.2.2.3. Unusual sting reaction

Such reactions have been observed with symptoms of neurological or renal disease, vasculitis, thrombocytopenic purpura, and serum sickness-like syndromes [5, 18]. These reactions are very rare, and their pathogenesis is mostly unclear. The initial manifestation of cold urticaria after wasp stings has also been described [19, 20]. 

### 2.3. Epidemiology of large local and systemic Hymenoptera sting reactions 

Data on the incidence of SAR to Hymenoptera stings vary widely depending on the population studied. In the United States [21] and in Europe [22] ~ 3% of the general population report SAR after Hymenoptera stings. There are significant national differences, and subpopulations, such as beekeepers and their family members, reporting systemic sting reactions much more frequently. In German-speaking countries, Hymenoptera stings are the most common cause of anaphylactic reactions in adults, whereas such stings are of secondary importance for anaphylaxis in children and adolescents [4, 23, 24, 25]. 

In a review of several studies, on average a quarter (18 – 42%) of the SAR induced by Hymenoptera stings in adults are severe (grade III or IV) [26]. In children, the proportion of moderate to severe reactions has been reported to be between 10% [27] and up to 20% [28, 29]. 

In Germany, between 2015 and 2019, 16 – 29 deaths from bee, vespid, or hornet stings were recorded annually by the Federal Statistical Office, almost exclusively in adults, mostly men [https://www.destatis.de/DE/Themen/Gesellschaft-Umwelt/Gesundheit/Todesursachen/]. The actual frequency of fatal sting reactions is likely to be higher, as anaphylaxis may be overlooked in sudden deaths or is difficult to diagnose with certainty post mortem. 

The frequency of large local reactions has been reported to be between 2.4% and 26.4% in the general population and up to 38% in beekeepers, depending on the population studied and the methodology used [5, 22]; in a recent study from Germany it was 4.8% [30] and in Austria 4.6% [31]. In children, the frequency of large local reactions can vary between 5.2% [32], 5.8% [28] and 9% [29]. 

## 3. Diagnosis 

### 3.1. Indication for diagnostic procedures 

The aim of allergy diagnosis is to classify the severity of a history of systemic allergic sting reactions and to assess the patient‘s individual risk of anaphylaxis. If an indication for venom immunotherapy (VIT) is considered on the basis of a suggestive history of a SAR due to an insect sting, evidence of IgE-mediated Hymenoptera venom sensitization (HVS) should be obtained and the offending insect identified on the basis of history and test results. 

In the general population, HVS without clinical relevance is common: ~ 40% of the total population and up to 50% of children show HV sIgE in serum, and only in a fraction of these cases there is evidence of true pathogenic, i.e., allergic, reactions [25, 30, 33, 34, 35, 36]. In the German Adult Health Study (DEGS), sIgE to bee and/or *Vespula* venom was found in ~ 23% of representatively selected subjects [37]. HV sIgE is also detectable in the serum of ~ 85% of cases with large local reactions [6]. 

In patients without a history of anaphylactic reaction (“diagnostic exclusion of allergy”), allergy tests are not indicated and should generally not be performed. The detection of a clinically irrelevant HVS may cause considerable uncertainty in the patient. In the case of a history of poisoning by numerous insect bites, an allergy test should also not be carried out. Exceptions may be made if the trigger (bee or vespid venom) needs to be identified, for example because of large local reactions. In such cases, the diagnosis should allow the physician to plan prevention measures to avoid such reactions. 

HV sIgE should be determined at the time of the patient‘s first presentation, even if this is shortly after the sting. If the result is negative, the measurement shall be repeated ~ 2 – 6 weeks after the sting. A marked change in the concentration of HV sIgE may indicate previous allergen exposure and the type of venom involved [38, 39]. In case of presentation > 2 weeks after the sting reaction, the tests should be performed as soon as possible, as a more or less rapid decrease of HV sIgE may occur depending on the individual atopy status [39, 40]. 

In the case of anaphylaxis after an insect sting, if sensitization cannot be demonstrated by either skin testing or HV sIgE (directed against the whole venom and venom components), and if a relevant clinical decision depends on such evidence (especially the indication for VIT), the tests should be repeated and additional investigations should be performed (see below). 

The aim should always be to diagnose the HV that has presumably caused the reaction. In the case of a reaction to a bumblebee sting, if appropriate test reagents are not available due to high cross-reactivity, tests with bee venom may be performed, and tests with *Vespula* venom may be performed in the case of a reaction to a hornet sting [41] (Box 1). 

### 3.2. Medical history 

Recordings of the medical history should include the number, symptoms, and course of the sting reaction, the situation in which the patient was stung, information provided by the patient on the type of insect, and individual risk factors for severe anaphylaxis. 

**3.2.1. Clinical symptoms**

In most cases, patients are not seen by an allergist in the acute phase, and symptoms are recorded by history. If medical notes or records of emergency treatment of the acute reaction are available, these should be taken into account. A questionnaire-based history is recommended (Table 4). 

Subjective or objective clinical signs or symptoms on the skin are helpful to classify a reaction as anaphylactic (see also 2.2.2.1). Typical subjective symptoms are pruritus of the palms, soles, scalp, and genitalia, and an ascending sensation of warmth and pressure over the ears; objective signs are flushing, generalized urticaria, or angioedema distant from the site of the sting. Particularly in the absence of cutaneous symptoms, it can sometimes be difficult to distinguish anaphylaxis with moderate circulatory symptoms (dizziness, tachycardia, pallor) from an anxiety response or vegetative pain reaction. 

The severity of the individual reaction should be classified on the basis of the clinical symptoms. The classification according to Ring and Messmer [7] (Table 3) has proved to be useful; the classification according to Mueller [42] has been widely used, especially in English-language publications. 

Possible differential diagnoses should be considered when taking the patient‘s history: insect stings to the head or neck can cause marked local reactions that may clinically correspond to angioedema without a systemic reaction being present. Exclusively subjective complaints (e.g., anxiety, palpitations, feeling of faintness) immediately after the sting may indicate a psycho-vegetative reaction. Other differential diagnoses of anaphylaxis should be considered [43], as well as the possibility that the anaphylaxis was caused by a trigger other than a bee or a vespid sting. The history should include other triggers that may have caused the reaction. 

**3.2.2. Culprit insect**

Clues to the culprit insect come mainly from the history of the circumstances of the sting (Table 5). In most cases, the patient can state that a bee or wasp has caused the reaction, but the distinction between bees and wasps is often unreliable. Stings by hornets, paper wasps (*Polistes*), yellowjackets (*Dolichovespula*) or bumblebees are rare events compared with stings by bees or *Vespula* species (e.g. *Vespula vulgaris* or *Vespula germanica*). However, stings from bumblebees, for example, are possible in horticulture because they are used for pollination in greenhouses, and stings are common there. 

**Figure 1.**
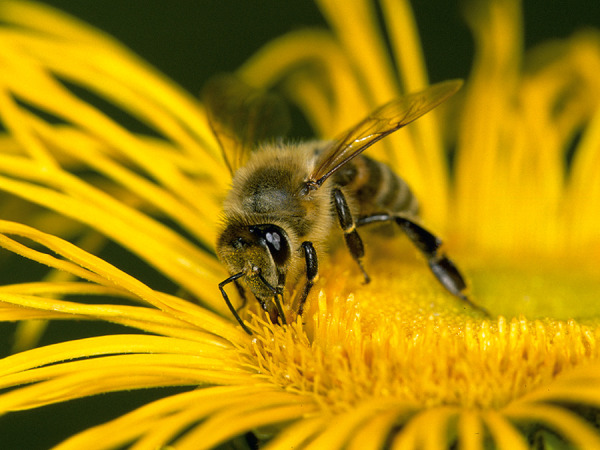
*Apis mellifera *(honey bee).

**Figure 2.**
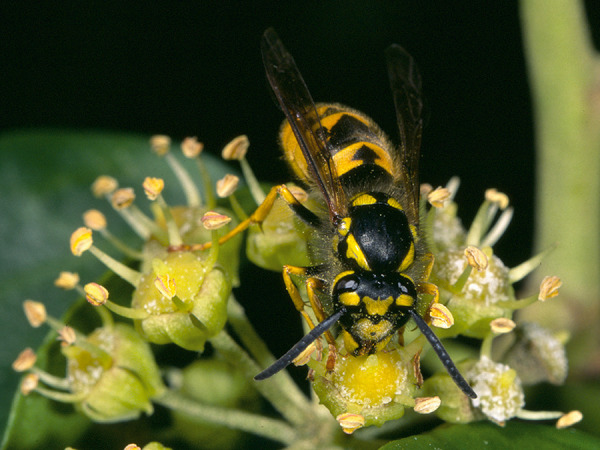
*Vespula germanica* on ivy.

**Figure 3.**
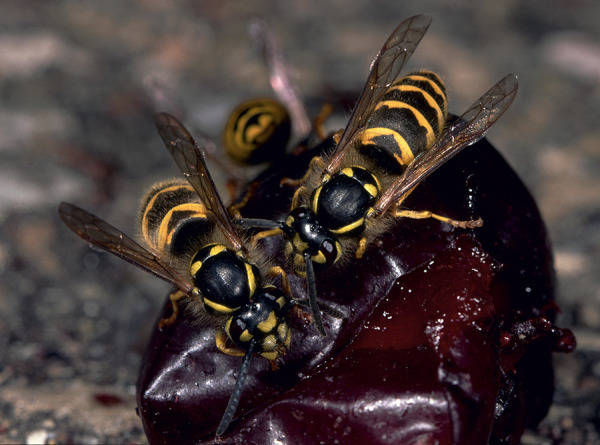
*Vespula vulgaris *on a plum.

**Figure 4.**
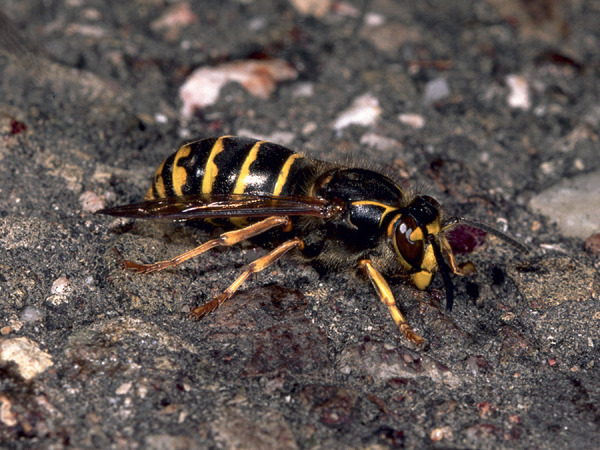
*Dolichovespula media* on the earth.

**Figure 5.**
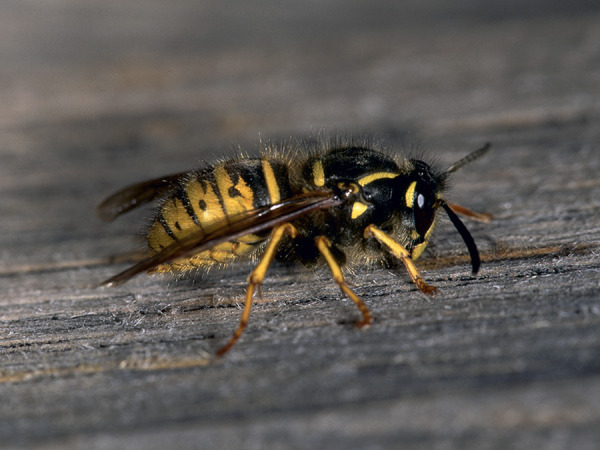
*Dolichovespula saxonica*.

**Figure 6.**
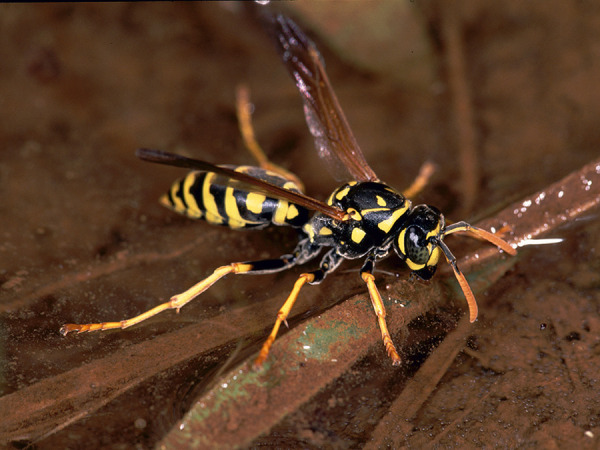
*Polistes dominulus* while drinking.

**Figure 7.**
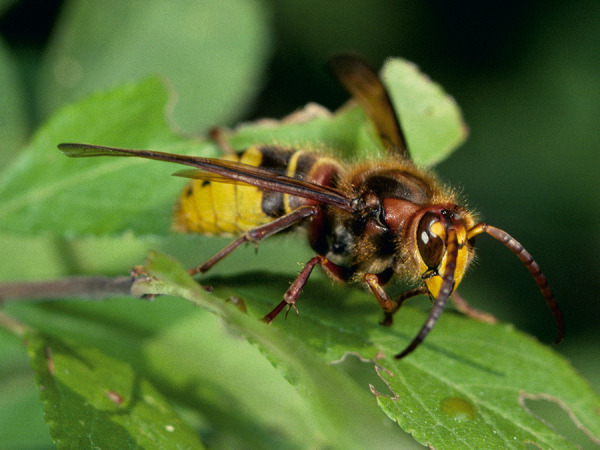
*Vespa crabro* (hornet) on a leaf.

**Figure 8.**
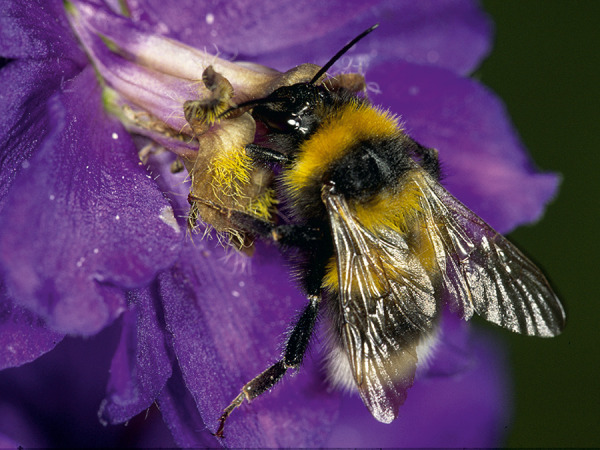
*Bombus hortorum* (bumblebee).

Some blood-sucking insects, such as horseflies, can also cause pain when they sting. Given that the majority of the general population cannot reliably distinguish between bees and vespids [45], and that some languages do not have separate names for the various Hymenoptera families and subfamilies (which is particularly relevant in the case of migrants), patients should be shown photographs of the suspected insects and, if necessary also dipterans, to check whether the suspected insect can be correctly identified (Figures 1, Figure 2, Figure 3, Figure 4, Figure 5, Figure 6, Figure 7, Figure 8, source: Volker Mauss). The history should also include questions about tolerated Hymenoptera stings before and after the index sting, as even tolerated stings can lead to sensitization, which must then be taken into account when interpreting the results. With regard to permanent tolerance, the history of a tolerated sting after an index sting is not reliable: the probability of developing anaphylaxis after another sting by the same insect is ~ 40%, even if there has been a previous allergic reaction caused by the same insect [46]. A tolerated sting does not rule out severe anaphylactic reactions to subsequent stings [47]. 

**3.2.3. Individual anaphylaxis risk**

An individually higher risk exists in the case of

- increased exposure to bees or wasps with the risk of more frequent stings (Table 6) or
- in the presence of patient-specific risk factors for very severe reactions.

Essentially, an increased risk of future sting exposure can be determined from the patient‘s medical history, so questions should be asked about, among other things, occupation, leisure activities, and time spent outdoors (Table 6). In addition, questions should be asked about medications or specific factors (comorbidities) present at the time of the examination and at the time of the sting event, about the type of occupation and leisure activities associated with outdoor exposure, and about specific risk factors that increase the risk of being stung. 

The greatest risk factor for a more severe anaphylactic sting reaction is an elevated baseline serum tryptase concentration (bST) [9, 48, 49, 50] and/or mastocytosis [10]. Even without formal evidence of mastocytosis, very severe anaphylactic sting reactions in adults are associated with elevated bST [49, 51, 52, 53]. The bST-associated risk already increases when bST is below the 95^th^ percentile (11.4 µg/L) [10, 48]. Many patients with elevated bST are likely to have mastocytosis. bST > 20 µg/L is a minor diagnostic criterion for the diagnosis of indolent systemic mastocytosis [54]. However, mastocytosis cannot be excluded when bST is < 20 µg/L or even below the 95^th^ percentile (11.4 µg/L) [55]. Conversely, elevated bST may be found in other conditions, including hereditary α-tryptasemia, which may [56] or may not [57] be associated with mastocytosis. Hereditary α-tryptasemia is also associated with a risk of particularly severe anaphylaxis. 

In the case of mastocytosis and/or elevated bST (> 20 µg/L) in adults, there is a particularly high risk of a) the occurrence of HVA per se and b) particularly severe anaphylactic sting reactions.

- ~ 5% of adult patients presenting for evaluation of insect sting allergy are diagnosed with indolent systemic mastocytosis [58]. Data on patient sting provocation during VIT also show that ~ 5% of patients with HVA have concomitant mastocytosis [59]. Conversely, questionnaire-based surveys have shown that 25% of patients with mastocytosis report HVA [60].
- Grade III or IV anaphylaxis occurs in ~ 75% of patients with HVA and mastocytosis and/or elevated bST, whereas such severe reactions occur in less than 20% of patients without mastocytosis and/or elevated bST [59].

In children, systemic anaphylactic sting reactions have been shown to be associated with comparatively higher bST; however, the differences compared with controls are small, and bST may even be within the normal range [61]. Cutaneous mastocytosis in childhood is not a risk factor for the development of HVA [62]. 

The likelihood of severe sting anaphylaxis increases with age [48, 50]. When reactions were fatal, those who died were often of advanced age [26]. In contrast, children have a much better prognosis: although younger children are more likely to be stung again, they rarely have a severe SAR despite not having had VIT [63]. In general, children are less likely to have a severe SAR to subsequent stings [24]. 

Other potential patient-specific risk factors may exist; in assessing whether and, if so, what specific importance should be attached to them, it is important to note that these risk factors have been identified by retrospective observational studies involving predominantly adult patients. In the case of ACE inhibitors and β-blockers, for example, the way in which the results were obtained often makes it impossible to distinguish between a possible effect of the drug itself and effects of the underlying cardiovascular disease being treated. Multivariate analyses that included pre-existing cardiovascular risk factors found that they may independently increase the risk of severe anaphylaxis to insect stings [10, 50]. Overall, data on the association between sting severity and the use of β-blockers and/or ACE inhibitors are inconsistent: some groups have found a higher risk of severe reactions in HV-allergic patients taking ACE inhibitors or β-blockers [10, 48], while other groups could not confirm this [9, 50, 53]. Differences in the type of statistical analysis may explain this discrepancy. 

As ACE inhibitors also inhibit kininase and thus the degradation of bradykinins released during anaphylaxis, there is a pharmacological basis for a specific adverse effect in anaphylactic reactions, which has also been shown in animal studies [64]. 

Repeated stings over a period of time carry the risk of boosting and may lead to an increase in the severity of the reaction [10, 48, 65, 66]. The observation that men have a higher incidence of severe and even fatal reactions than women [48, 65] may reflect the higher risk of stinging associated with more frequent outdoor activity in men, which may increase the severity of a sting reaction through a booster effect of further stings (Box 2). 

**3.2.4. Hymenoptera venom allergy as a consequence of an occupational accident and as an occupational disease**

In Germany, a more severe sting reaction (anaphylaxis as well as a large local reaction) during an activity covered by statutory accident insurance may constitute a work accident or an occupational disease. In the case of a work accident involving an allergic reaction, there is an indication for acute treatment at the expense of the accident insurer, even if the HVA existed before the accident or was not acquired occupationally. 

However, if the HVA is verifiable as a consequence of the insured activity, all other indicated treatment measures (including VIT) are also indicated at the expense of the accident insurer. However, if the allergic reaction is a consequence of HVA and fulfils the criteria of an occupational disease (in German: Berufskrankheit (BK)) No. 4301 and/or 5101 Ordinance on Occupational Diseases (in German: Berufskrankheitenverordnung (BKV)), administrative processing as an occupational disease has priority. In this case, all necessary preventive and therapeutic measures may be taken at the expense of the statutory accident insurance within the framework of §3 BKV in order to prevent the development, aggravation or recurrence of an occupational disease by all appropriate means [67]. 

### 3.3. Determination of Hymenoptera venom-specific IgE antibodies 

**3.3.1. Whole venom**

Initially, serum sIgE testing against bee and *Vespula* venom and, if necessary, other HV should be performed. For pragmatic reasons, the determination of sIgE against whole bee and *Vespula* venom may be omitted and only molecular allergy diagnostics performed. If a stepwise approach is planned, it would be optimal to perform the extended diagnostics from the same serum sample and therefore freeze the supernatant of the sample used for the first diagnostics (allowing further measurements to be performed from it). Several commercial assays based on an automated ELISA (enzyme-linked immunosorbent assay) procedure are available for the detection of sIgE. The methods differ in, among other things, the type of solid phase to which the allergen extract is coupled, the instrumental set-up, and the degree of automation of the assay. Currently available methods for the detection of sIgE vary in sensitivity and specificity. As results may vary depending on the method used, the method used should be specified when reporting results. 

Often, the concentration of sIgE to the pathogen increases significantly several days to weeks after a reaction as a result of boosting by antigen exposure [39]. Thus, repeated determination of HV sIgE in the first days to weeks after the sting reaction may provide clues to the culprit insect. It should be noted that even tolerated stings can induce sIgE production [36]. 

**3.3.2. Components of the venom**

HVs contain several components that can induce sIgE formation (Table 7). A distinction is made between venom-specific components and components which, due to their homology, are cross-reactive with other insect venoms or are pan-allergens. In the case of double-positive sIgE detection to bee and *Vespula* venoms, there is the possibility of

- True clinical double sensitization to both venoms,
- Clinically irrelevant positive results of sIgE against cross-reactive carbohydrate determinants (CCD), which are widespread in animals and plants [68, 69],
- Cross-reactivity of sIgE to homologous allergens present in both venoms, such as hyaluronidase, dipeptidyl peptidase IV or vitellogenin [68, 70].

Screening for the detection of sIgE to CCD in patient serum can be performed using CCD-rich substrates such as MUXF (CCD component from pineapple bromelain) or, alternatively, horseradish peroxidase. 

**Figure 9.**
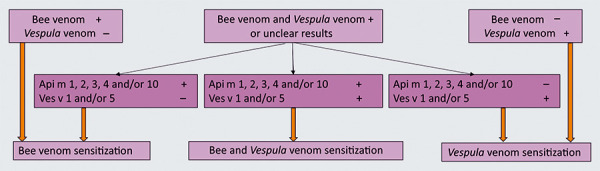
Stepwise diagnosis using whole venoms (bee venom (BV) and Vespula venom (VV)) and allergen components of bee venom (Api m) and Vespula venom (Ves v).

Especially in the case of double sensitization (i.e., detection of sIgE to bee and *Vespula* venom), we recommend extended sIgE diagnostics using recombinant HV components that do not carry CCD side chains that interfere with the diagnosis. Detection of sIgE to Api m 1, Api m 2, Api m 3, Api m 4, or Api m 10 indicates primary sensitization to bee venom [71], and detection of sIgE to Ves v 1 or Ves v 5 [71, 72] indicates primary sensitization to *Vespula* venom (Figure 9). 

If an allergy to other insects cannot be excluded, it should be noted that within the bee (Apidae) or Vespidae families there is a similarity of venom components at the molecular level. Depending on the individual sensitization profile, there may be only partial cross-reactivity to honey bee and bumblebee venom [41] or to venom from *Vespulae*, wasps (*Dolichovespula* spp.) or paper wasps (*Polistes*) (Box 3). 

**3.3.3. Additional in-vitro tests**

In the case of double sensitization to bee and vespid venom, or if a false negative result for the causative venom is suspected, cellular tests can be performed. These cellular tests are complex and are therefore reserved for specialized allergy centers. Some patients do not respond to these tests despite the presence of a relevant HVA, and interpretation of the individual dose-response curve requires experience with the test procedure. 

The principle is based on the fact that after in-vitro stimulation with the allergen, sIgE bound to the surface of peripheral blood cells leads either to cell activation with upregulation of activation markers (e.g., CD 63) or to the release of substances (e.g., leukotrienes or histamine) that can be detected by appropriate assays. In this way, HVS can be detected indirectly. Primarily, the basophil activation test (BAT) is the test of choice, which is also the best evaluated [40, 73, 74, 75, 76]. Other tests (leukotriene release test, cellular antigen stimulation test (CAST) or histamine release test) are hardly available at present and are unlikely to be available in the future due to the European requirements for standardization of in-vitro diagnosis (In-Vitro Diagnostic Regulation). 

Specific IgG antibodies to HV may be pathophysiologically relevant in patients with serum sickness-like or other unusual sting reactions. Determination of these antibodies may be useful in patients with such clinical entities, but is not relevant for the indication of VIT. A high concentration of HV-specific IgG antibodies is an epiphenomenon of allergen exposure, including immunotherapy, but does not prove protection against future systemic sting reactions [77, 78] (Box 4). 

**3.3.4. Baseline serum tryptase concentration**

In adults and children with a SAR to a Hymenoptera sting, if the reaction extends beyond the skin (i.e., ≥ grade II according to Ring and Messmer [7]), bST should be determined by a commercial assay using the 95^th^ percentile (11.4 µg/L according to the manufacturer‘s instructions) as the upper normal value. 

Skin examination, Darier test (rubbing a skin lesion suspected of mastocytosis to produce a wheal) and determination of bST should be used to diagnose cutaneous mastocytosis. In the case of cutaneous mastocytosis and/or elevated bST (> 20 µg/L) in adults (with suspicion of systemic mastocytosis), further diagnosis should be made; reference is made to the mastocytosis guideline [Hartmann et al. Mastocytosis, guideline of the DGAKI and the DDG, in preparation] (Box 5). 

### 3.4. Skin tests 

A skin test may be omitted if there is a particular risk associated with the skin test, if its performance would severely affect the patient, and/or if a clear result has already been obtained from in-vitro tests. Skin testing should be performed if the sIgE diagnosis is negative or if there is a discrepancy between the history and in-vitro findings. 

Skin testing [79] is performed with bee and *Vespula* spp. venoms, positive and negative controls. If necessary and if available, additional tests with other venoms are performed. The intradermal test is more sensitive than the prick test but more painful. 

A prick test with venom concentrations of 10 µg/mL, 100 µg/mL, and 300 µg/mL has been found to be useful in determining the threshold of reaction, although it should be noted that irritative reactions often occur at a test concentration of 300 µg bee venom [80]. If there is no reaction, an intradermal test can be performed with a final concentration of 1 µg/mL. Intradermal tests are usually performed with concentrations of 0.1 and 1.0 µg/mL in children, and additionally with 0.001 and 0.01 µg/mL in adults [81]. 

Simultaneous skin testing at all concentrations has been shown to be safe in a monocentric study [81]. To be on the safe side, sequential intradermal testing with increasing HV concentrations at ~ 15-minute intervals may be recommended in patients with a very severe sting reaction or at high risk. It should be noted that higher concentrations in both the prick test and the intradermal test may cause false positive reactions which must then be interpreted critically (Box 6). 

### 3.5. Sting provocation 

Sting provocations (see also 5.5.) can only be offered at specialized centers and according to the local resources. For logistical and infrastructural reasons, this diagnostic tool is not a diagnostic standard that is generally available. 

If sting provocation with a live insect is possible, it should only be performed in patients on tolerated maintenance VIT, because – in contrast to provocation with food or drugs – the provocation dose cannot be increased gradually and thus there is a risk of difficult-to-control, sometimes life-threatening reactions [82]. In addition, the absence of a systemic reaction from a single sting provocation is not reliable. For example, it has been shown that after an initially tolerated sting in adult patients with a history of an anaphylactic sting reaction, a further sting led to a new systemic reaction in ~ 20%, which was severe in nearly half of them [47]. Even after completion of VIT, sting provocation should not be performed, as this may lead to a „booster“ causing a reactivation of the allergic reaction state (Box 7). 

### 3.6. Evaluation of diagnostic results 

Several variables must be taken into account in the interpretation of diagnostic results:

- Patient differentiation between bee and vespids is often unreliable.
- False negative and false positive results are possible with all test systems.
- The time course of sensitization parameters in relation to stings (including tolerated stings) must be considered. After stings, there is often an increase in the concentration of sIgE in the serum within a few weeks [36]. This increase is usually followed by a long-term decrease, even to levels below the detection limit [39, 83, 84].
- In the general population, HVS is common (> 40%) in the absence of a history of clinically relevant SAR to stings [30]. Patients with high total IgE levels are more prone to clinically irrelevant HVS [85].
- There is no correlation between the degree of sensitization at the time of diagnosis and the severity of previous anaphylactic reactions [48].

Despite extensive testing and critical evaluation of the results, it is not always possible to identify a specific venom when planning therapy. 

## 4. Symptomatic therapy and general measures 

### 4.1. Local reactions 

**4.1.1. Acute therapy**

Acute local reactions can be treated with a cool, moist compress left on for ~ 20 minutes, repeated once or twice at intervals of several hours. For stings on the arms and legs, immobilization and elevation may also help reduce swelling. The external application of glucocorticoids (in cream or gel form) or the use of an antihistamine is often practiced without evidence from comparative trials. The same applies to the use of over-the-counter hyperthermia devices advertised for this purpose. 

In the case of a large local reaction, especially in the case of relevant functional limitations of the hands and feet, early and short-term systemic glucocorticoid therapy (0.5 – 1 mg prednisolone equivalent/kg body weight PO) and oral application of a non-sedating H1-receptor blocking antihistamine may be justified. In contrast to mosquitoes (Diptera), pathogen transmission does not play a role in Hymenoptera. Antibiotics are not indicated for the treatment of non-infectious lymphangitis or lymphadenopathy. 

If the sting has occurred in the upper respiratory tract, prophylactic systemic antiallergic therapy (H1-blocking antihistamine and systemic glucocorticoid) and medical follow-up should be given because of the possibility of severe swelling leading to potentially life-threatening airway obstruction (Box 8). 

**4.1.2. Long-term management of large local reactions**

Patients with a history of a large local reaction may be prescribed on-demand medication (topical glucocorticoid in a cream or gel base and, if necessary, a systemic glucocorticoid and an H1-blocking antihistamine) together with instructions on how to proceed after a new sting. Even in the case of a previous unusual sting reaction, the need for systemic glucocorticoid administration and/or further symptomatic therapy should be considered immediately if a new sting occurs. If necessary, the patient should seek additional medical advice to discuss any current contraindications to systemic glucocorticoid therapy and to determine the duration of therapy (Box 9). 

### 4.2. Non-specific general symptoms 

Non-specific general symptoms can be alleviated symptomatically with non-steroidal anti-inflammatory drugs. Psycho-vegetative reactions (e.g., hyperventilation) primarily require situational reassurance. In practice, this reaction is difficult to distinguish from anaphylaxis in the acute situation, even for experienced emergency physicians. This important differential diagnosis is more likely to be made retrospectively, taking into account the patient‘s experience in other situations. 

Psycho-vegetative reactions and non-specific general reactions are not indications for VIT. 

### 4.3. Systemic allergic reactions 

**4.3.1. Acute therapy**

A SAR should be treated according to the severity and the guideline for the acute management of anaphylaxis [43]. 

In the case of unusual sting reactions (e.g., sting-associated immune complex vasculitis), systemic administration of a glucocorticoid is usually the basis of therapy, with further symptomatic treatment. In the case of clinical signs of venom poisoning (e.g., hemolysis, rhabdomyolysis) after multiple stings, symptomatic supportive therapy is indicated, including intensive care, if necessary. 

**4.3.2. Long-term management of systemic allergic reactions**

Patient education on avoidance and self-medication and counseling on the diagnosis and possibility of VIT should be given immediately after anaphylaxis or before discharge from inpatient monitoring. Patients with a history of a SAR after a Hymenoptera sting require long-term management consisting of

- allergen avoidance,
- self-help measures in the event of a new sting and, when applicable,
- reduction of risk factors, and
- VIT.

4.3.2.1. Allergen avoidance

The patient should be given verbal information about measures to avoid Hymenoptera stings; a leaflet can also be given (Table 8). 

In the case of increased occupational exposure associated with a risk of more severe anaphylaxis, the patient should be prevented from the hazardous activity during the insect flight period by being transferred to a non-exposed working place within the company and, if necessary, by certification of incapacity to work until VIT has been initiated. If the patient is particularly at risk (e.g., grade IV anaphylaxis, diagnosis of mastocytosis), a sting provocation may be considered in individual cases before resuming work to ensure the onset of the clinical protective effect of VIT. 

4.3.2.2. Self-help measures in case of re-stings

The patient should be informed of what to do in the event of being stung again and given instructions on how to use the emergency kit (Table 9), if prescribed. A suggested patient information leaflet is shown in Table 10. Any person prescribed an emergency kit containing an adrenaline auto-injector (AAI) for HVA, should also receive a recommendation for VIT. When prescribing an emergency kit, it should be remembered that carrying the kit may mean a reduction in quality of life [86]. For some patients, the prospect of no longer having to carry the emergency kit with an AAI is a motivation to perform VIT. 

The following patients should seek immediate medical attention after a new sting

- Patients with a SAR right after an insect sting
- Patients who have not received VIT but are at high risk of severe anaphylaxis (mastocytosis, history of very severe sting reaction)

Participation in anaphylaxis training should be recommended to patients who are at high risk of future severe anaphylaxis, who are at high risk or who have developed a strong fear of such reactions. In Germany, in contrast to Austria, there is a well-established training concept for this, although training opportunities for adults are limited and the costs must either be borne by the patient or, in the case of statutory health insurance, an application must be made in advance for the costs to be covered. Free online training is available from the German Allergy and Asthma Association Federation (https://www.daab.de/termine/online-seminare/anaphylaxie-online-seminare/). 

**Emergency kit**

An EAACI expert group has worked intensively on the indication and composition of the necessary emergency self-medication for patients suffering from insect venom allergy [86] and has formulated recommendations for the prescription of an AAI before, during, and after VIT. These recommendations have also been incorporated into the European guideline for the treatment of insect sting allergy [4] and the German guideline for the acute treatment and management of anaphylaxis [43]. If there is a plausible history of HVA, the indication for an emergency kit with an AAI depends on whether VIT has already been started or successfully completed and whether there are anamnestic risk factors for severe anaphylaxis after insect stings, for treatment failure of VIT, or for increased sting exposure (Table 6). Even with a history of mild SAR (grade I), an AAI may be prescribed after individual consultation with the patient, especially if there is a high risk of re-exposure. An AAI may not be prescribed if the risk of a subsequent systemic sting reaction is approximately comparable to that of the normal population [4]. This can be assumed in the case of successful VIT and a tolerated sting reaction either after a field sting or after a sting provocation. 

Whether an AAI can be omitted in certain groups of patients who have already reached the maintenance dose of VIT is judged differently [86]. Considering that most patients are already protected at this point [87], the authors of the EAACI guideline on insect sting allergy have recommended that in the case of mild to moderate SAR (severity grade I – II) and in the absence of additional risk factors, the prescription of AAI may be discontinued once the maintenance dose has been reached [4]. After successful completion of VIT (maintenance dose achieved without complications, regular and well-tolerated maintenance therapy), an AAI may be omitted in patients who have developed only moderate systemic symptoms (grade II) and who have no additional risk factors for non-response to VIT (see 5.6.) [86]. If severe anaphylaxis (grade III or IV) was present initially, or if there are additional risk factors for non-response to immunotherapy (see below), the emergency kit should continue to be carried during the insect flight season even after VIT has been completed. 

The procedure for prescribing an AAI in adults and children is basically the same, but there are more risk factors for severe anaphylaxis in adults overall. Depending on the individual risk profile, the indication for an AAI is more common in adults. In contrast, children usually do well after VIT and the risk of severe anaphylaxis is low [24, 63, 88]. However, the risk of anaphylaxis also increases slightly with age in children [24, 63], and children who initially had severe anaphylaxis also have a higher risk of recurrent anaphylaxis both during and after VIT. Another risk factor for recurrent systemic reactions is the behavior of the children: for example, the risk of anaphylaxis increases with sporting activity after the sting. Table 11 summarizes the absolute and relative indications for prescribing AAI in insect sting allergy. 

Two AAIs should be prescribed in cases of [43.

- History of particularly severe anaphylaxis
- High body weight: > 100 kg
- Uncontrolled bronchial asthma
- Poor accessibility of the nearest medical emergency service
- Particularly high risk of severe anaphylaxis (e.g., adults with mastocytosis)
- Organisational: for nursery/school and according to family situation

If there is an increased risk of adrenaline side effects (e.g., severe cardiovascular disease), the indication for self-administration of adrenaline should be reviewed by a cardiologist (Box 10). 

4.3.2.3. Handling of risk factors for severe allergic sting reactions

Several potential risk factors for severe anaphylaxis, such as age, sex, or mast cell disease (mastocytosis, elevated bST), previous severe sting reactions cannot be modified. In essence, therefore, non-modifiable risk factors are a major reason for performing VIT. Only in the case of certain drugs, the risk can be modified. 

**β-blockers and ACE inhibitors**

Typically, β-blockers and ACE inhibitors are used to treat cardiovascular disease, which in turn is a risk factor for more severe anaphylaxis. Because cardiovascular disease is more important than the comparatively rare systemic allergic sting reactions, appropriate management of cardiac disease is a priority, even though β-blockers or ACE inhibitors may adversely affect the course of an anaphylactic reaction. ACE inhibitors used to treat arterial hypertension can usually be replaced by other agents. In heart failure, they should be continued because, unlike angiotensin receptor antagonists, they reduce mortality in heart failure [89]. If discontinuation is not possible, appropriate treatment of HVA becomes more urgent (Box 11). 

## 5. Allergen-specific immunotherapy 

Robust scientific evidence for the efficacy of VIT is limited [3], with few randomized, placebo-controlled [90] or whole body extract-controlled [91] trials demonstrating efficacy. A dose-dependent and Hymenoptera venom-specific effect of VIT has been demonstrated in reviews of observational studies [46] and large case series [59, 87, 92, 93, 94, 95]. Therefore, despite the lack of randomized trials, the efficacy of VIT is highly probable, and the conduct of future randomized, placebo-controlled trials is ethically questionable. 

A high percentage of patients can be protected from recurrent systemic sting reactions, at least for the duration of VIT. Pooled data from sting provocations performed using a 100- to 200-µg maintenance dose show an efficacy of 82 – 95% for bee VIT and 96 – 99% for *Vespula* VIT [46, 59, 94, 96]. In addition to providing clinical protection, VIT significantly improves patients‘ quality of life [92, 93, 95]. 

The guideline on AIT in IgE-mediated allergic diseases [97] should also be followed when treating HVA. Patients should be informed prior to treatment that VIT usually needs to be continued for 3 – 5 years and that early discontinuation may have a detrimental effect on the disease. In addition, the instructions for use for each insect venom product should be followed; these may differ from the recommendations in this guideline. 

### 5.1. Indication 

The recommendations in this guideline are consistent with the current recommendations of the EAACI [4]. Accordingly, the indication for VIT in adults is for

- patients with a history of grade ≥ II anaphylaxis to bee or vespid stings
- patients with grade I systemic sting reactions with risk factors or quality of life impairment due to HVA
- and with evidence of IgE-mediated sensitization (as determined by skin testing and/or HV-sIgE concentrations or positive cellular test results) to the offending venom.

Similarly, in an update on stinging insect hypersensitivity, the American Academy of Allergy, Asthma and Immunology recommends VIT only for sting reactions of severity grade ≥ II unless there are special considerations such as risk factors for severe anaphylaxis, increased exposure, and decreased quality of life [21]. As a previous mild SAR to a sting is a risk factor for subsequent more severe sting anaphylaxis in adults [48, 65, 66], VIT may also be recommended for adult patients with increased exposure if there has only been a grade I reaction. In any case, VIT is recommended for all adults with risk factors for severe anaphylaxis, regardless of the severity of the previous SAR. 

In the absence of evidence of IgE-mediated sensitization, VIT should not be performed, except in patients at high risk of severe anaphylaxis (especially mastocytosis, cardiovascular or respiratory arrest in previous anaphylaxis). In these patients, the offending venom cannot be identified with certainty, and treatment with both venoms should be considered. In the case of large local reactions, there is no indication for VIT. 

**Figure 10.**
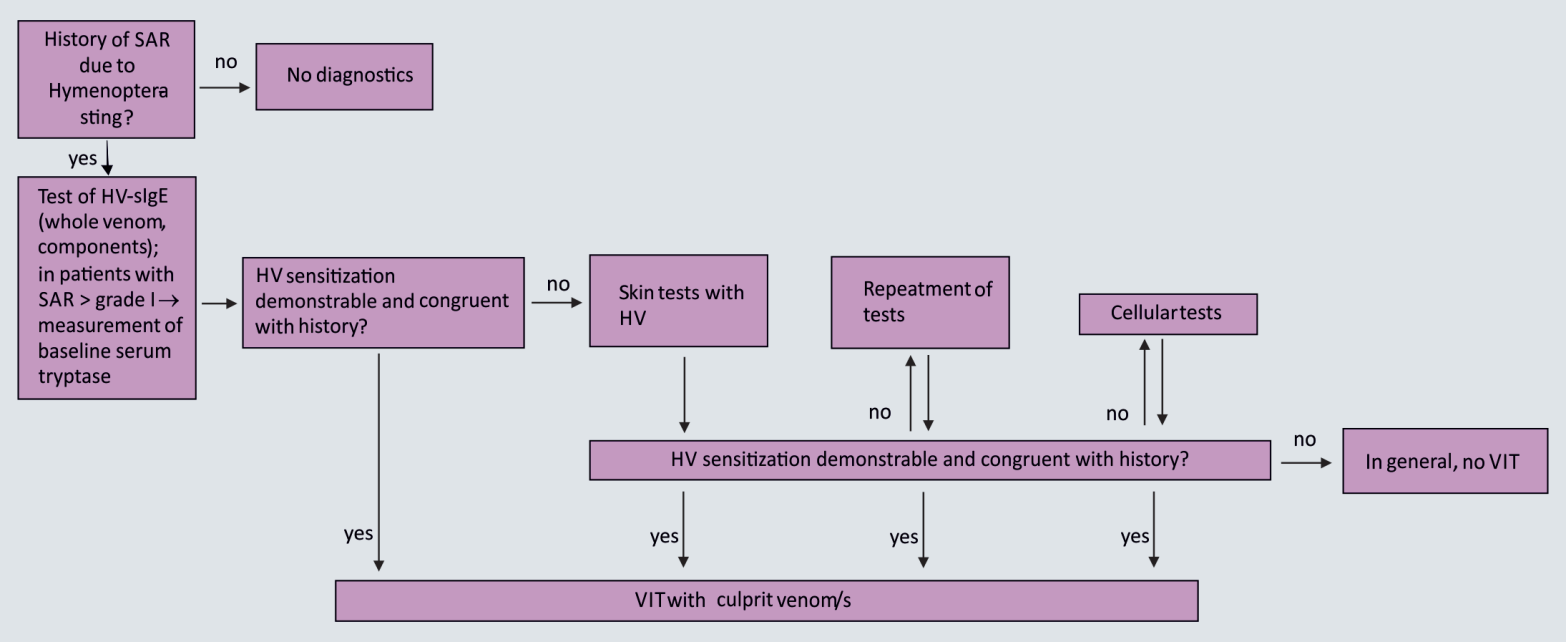
Algorithm for the diagnosis of suspected Hymenoptera venom allergy.

The indication for VIT with bee or vespid venom is shown as an algorithm in Figure 10 (Box 12). 

**5.1.1 Specifics for children**

According to an EAACI position paper, VIT is absolutely contraindicated in children under 2 years of age and relatively contraindicated in children aged 2 – 5 years. This recommendation is based on the fact that there is a paucity of efficacy and tolerability data in this age group. 

An observational study in children aged 2 – 16 years with a history of grade I systemic allergic sting reactions showed that subsequent stings – even without VIT – led to SAR in less than 20% of children, and these were again only mild [98]. Even in children with grade I – II reactions who were not treated with VIT, only mild reactions, if any, were observed with subsequent stings [24, 99]. It should be noted that the published data were collected retrospectively and the (prognostically less reliable) outcome was the reaction to field stings. Therefore, VIT may not be necessary in children with mild reactions limited to the skin. However, therapeutic approaches should be discussed and defined with the child and/or caregivers on an individual basis, taking into account the reliability of the medical history, the quality of life, and possible risks from environmental exposures and the child‘s behavior. 

Another aspect to consider when deciding for or against VIT in children and adolescents is the protective effect of VIT that lasts into adulthood: 13% of patients who experienced a grade I reaction as a child had SAR to repeated stings during a mean follow-up of 18 years without VIT. This rate dropped to 0% if the patients had received VIT [99]. 

### 5.2. Contraindications 

The recommendations of this guideline, the EAACI guideline on VIT [4], and an EAACI position paper on contraindications to AIT with aeroallergens or insect venom allergens [100] differ in several respects from the contraindications listed in the manufacturers’ product information. For example, severe reactions caused by VIT requiring adrenaline are very rare, whereas in patients with severe cardiovascular disease who are not protected by VIT, the risk of severe anaphylaxis after a field sting is much higher. 

Beyond case reports, there is no evidence to support the relevance of some of the conditions listed in the product information as potential contraindications to VIT. These conditions include aggravation of the underlying disease by concomitant VIT or, conversely, poor tolerance of VIT by the underlying disease. Patients should be informed about possible interactions (e.g., attenuation of adrenaline effects by β-blockers), and this information must be documented by written informed consent prior to VIT. 

Temporary contraindications to VIT (Table 12) apply as for treatment with aeroallergens [97]. In addition, VIT should not be used in acute infectious diseases, such as influenza, or in close temporal relation to vaccination against infectious agents. The presumed contraindication may either be stabilized by appropriate therapy or resolve spontaneously over time. VIT should only be performed subsequently. 

Whether contraindications such as malignant tumors argue for a permanent omission of VIT is subject to a risk-benefit assessment in each individual case. There is no evidence that VIT worsens malignant disease [101]. In patients with malignant disease, VIT is performed in consultation with the treating oncologist, taking into account the individual prognosis, risk of metastasis, and chance of remission; if possible, the phase with the highest risk of relapse should be over and the patient should be in a state of remission. If complications of the malignancy occur, VIT is usually discontinued temporarily or permanently. 

Given the good tolerability of VIT, severe cardiovascular or pulmonary disease will primarily increase the risk of anaphylaxis after a field sting in those patients who are not protected by VIT. Optimal medical management of the underlying disease is required before initiation and during continuation of VIT. Prospective monocenter [9] or multicenter [53, 102] studies on the risk of β-blockers and/or ACE inhibitors during VIT did not find an association between these medications and the incidence of VIT adverse events. 

In the case of autoimmune diseases, a distinction must be made between organ-specific diseases (e.g., Hashimoto’s thyroiditis, ulcerative colitis and Crohn‘s disease, type 1 diabetes mellitus), which in principle do not represent a contraindication to VIT, and systemic autoimmune diseases (e.g., active systemic lupus erythematosus). Active systemic autoimmune diseases are considered absolute contraindications to VIT [100]. 

Pregnancy is a contraindication to the initiation of VIT. VIT tolerated prior to pregnancy can be continued during pregnancy. If this can be planned, VIT should be started before the onset of pregnancy in women of childbearing age. This will protect the unborn child from the consequences (abortion) of SAR that may develop in the mother after an insect sting [103, 104]. 

In patients with congenital or acquired immunodeficiencies or on immunomodulatory therapy, a reduction in the efficacy of VIT can be expected but may depend on the disease or the intensity of disease-specific therapy (e.g., > 15 – 20 mg prednisolone equivalent). Specific clinical trials on the efficacy of VIT in patients concomitantly taking immunosuppressive or immunomodulatory drugs are lacking. However, information is available from studies of patients receiving different types of vaccinations at the same time. Because of various immunological similarities, this information can be applied to VIT, which is also called “allergy vaccination” [105]. For example, vaccinations are considered effective even when combined with long-term systemic administration (> 4 weeks) of less than 20 mg prednisolone equivalent/day [106]. Similar observations have been made for methotrexate, tumor necrosis factor-α inhibitors (but not for their co-administration), and hydroxychloroquine, although there is no general information on their dosage [107, 108]. 

There is also limited data on the concomitant use of immunomodulatory drugs, such as biologics, and VIT. From an immunological point of view, therapeutics that inhibit the Th2 immune response may even have a supportive effect [109, 110]. For example, the anti-IgE antibody omalizumab may reduce the risk of VIT-related anaphylactic reactions in patients at risk. A recently published study on the concomitant use of dupilumab and an AIT in patients with grass pollen allergy suggested a corresponding reduction, while the clinical efficacy of VIT remained unaffected [111]. Several studies on the efficacy of COVID-19 vaccines in patients receiving different biologics did not show any adverse effects [112]. 

Finally, the initiation or continuation of VIT in patients taking immunosuppressive or immunomodulatory drugs is certainly possible but will depend on an individual risk-benefit assessment. 

Active HIV infection is an absolute contraindication to VIT. In the case of a drug-controlled HIV infection, VIT should be performed if indicated (Box 13). 

### 5.3. Practical aspects of Hymenoptera venom-specific immunotherapy: patient education, updosing 

Prior to initiation, an appropriate information sheet (see Therapy Information in [97]) is used to explain the possible risks and side effects of VIT, recommendations on how to avoid them, and what to do if problems occur. Written informed consent must be obtained from the patient or the patient‘s guardian. 

Before each injection, an orienting medical history is taken of any contraindications to VIT that may have arisen in the meantime and, in the case of outpatient therapy, and if applicable, a check is made to ensure that the emergency kit has been taken along. 

The allergen is administered by subcutaneous injection. There are different protocols for initial dosing, examples of which are shown in Table 13. The standard maintenance dose is 100 µg HV per injection. As efficacy is dose-dependent [59, 96, 113] and bee venom therapy is generally less effective than vespid venom therapy [59, 96], adult patients with bee venom allergy and the presence of risk factors may be treated a priori with an increased maintenance dose of 200 µg. This is the case, for example, for mast cell disease or for those with intensive exposure, such as beekeepers with bee venom allergy, who should receive VIT with an increased maintenance dose from the outset. A protocol for increasing the usual maintenance dose from 100 µg to 200 µg is given in Table 14. 

In patients with vespid venom allergy, an increased maintenance dose from the start may also be indicated if the risk of severe anaphylaxis is unusually high. In children, there is insufficient experience to support an increased maintenance dose and procedures should be determined individually (Box 14). 

**5.3.1. Selection of the venom**

The choice of venom for VIT is based on the overall results of the diagnostic procedures. If both bee and *Vespula* venom allergies are present, or in the case of simultaneous sensitization to both bee and *Vespula* venom, if it has not been possible to determine whether the sting was caused by a bee or a *Vespula* spp., both venoms should be used for treatment. This is especially true for high-risk patients. 

Anaphylaxis to bumblebee or hornet stings is very rare in the general population. As bumblebees are used for pollination in greenhouses, stings and subsequent SAR may be common in greenhouse workers. Furthermore, as patients are more at risk from exposure to cross-reacting bee or *Vespula* venoms, VIT should be performed with the related and partially cross-reacting venoms [114]. However, given the only partial cross-reactivity between bumblebee and bee venom [41], bumblebee venom [115] if available, should be preferred in cases of specific exposure. The same applies to allergy to hornet, yellowjacket, *Polistes*, or *Dolichovespula* venom. If there is reasonable suspicion of a primary allergy to bumblebee, hornet, *Dolichovespula*, or paper wasp venom, patients should be referred to a specialist allergy center for co-evaluation. If there is clear evidence of limited cross-reactivity, VIT with bumblebee, yellowjacket, paper wasp, or hornet venom may be initiated as an individual curative trial. 

At the time of writing, neither bumblebee nor hornet venom is licensed in German-speaking countries and can only be obtained from international pharmacies in other European countries as aqueous or tyrosine-adsorbed preparations (e.g., Anallergo, Italy). To date, *Polistes* venom has mainly played a role in the Mediterranean region, where it is commercially available from various suppliers (Box 15). 

**5.3.2. Choice of the therapeutic allergen**

Various manufacturers offer therapeutic allergens obtained by electrostimulation in the case of bee venom and by the preparation of venom sacs in the case of vespid venom. After preparation, different types of therapeutic allergens are produced:

- Aqueous extracts, which are available as less or highly purified therapeutic allergens.
- Highly purified HV preparations adsorbed on aluminium are licensed in German-speaking countries.
- Highly purified allergens adsorbed on tyrosine are available in other European countries.

In terms of local reactions, highly purified venom preparations, both aqueous and depot, are significantly better tolerated than less purified aqueous preparations [116, 117]. Sustained-release formulations adsorbed with aluminium hydroxide are also associated with a lower risk of SAR [118] but cannot be used for rapid updosing. However, they are suitable for slow updosing and maintenance therapy. Due to the addition of aluminium, if a higher dose (> 100 µg maintenance dose) is used and the duration of therapy is expected to exceed 5 years, the use of depot preparations is only possible in the context of off-label use or in combination with aqueous extracts. For a double VIT with the standard dose of bee venom and vespid venom, the use of a depot preparation is permitted. 

It has been shown that in some therapeutic bee venom preparations, possibly as a result of processing or storage, individual components such as Api m 3, Api m 5, or Api m 10 are absent or underrepresented [119, 120, 121] and that dominant sensitization to Api m 10 (> 50% of sIgE to bee venom is directed against Api m 10) is associated with an increased risk of treatment failure (according to a retrospective study) [121]. However, no prospective studies have investigated the extent to which sensitization to these components determines bee venom allergy and the extent to which the absence of these components in therapeutic bee venom preparations reduces the efficacy of specific immunotherapy with bee venom. 

**5.3.3. Dose-escalation phase**

There are many therapeutic protocols for the dose-escalation phase (Table 13), and only a few comparative prospective studies of their adverse effects and efficacy have been published [53, 102, 116, 122]. 

There are two fundamentally different approaches to the dose-escalation phase:

- Rapid hypersensitization (usually in hospitalized patients receiving an aqueous allergen preparation) with maintenance dose achieved after hours (ultra-rush) to a few days (rush)
- Conventional VIT (outpatients receiving an aqueous or aluminium hydroxide-adsorbed allergen preparation) with maintenance dose achieved after weeks to months, also as cluster protocols.

Both approaches have their pros and cons. In many, mostly monocentric, observational studies, faster updosing regimens have been reported to be well tolerated [94, 123, 124, 125, 126]. In comparative studies, slower updosing protocols have been associated with a lower risk of adverse events for common allergic reactions. For example, in a retrospective multicenter study of 840 patients [122] and in a prospective multicenter study of 680 patients [102], a faster updosing protocol was associated with a slightly higher incidence of SAR with VIT. 

The choice of protocol depends on regional treatment capacity, the potential side effects of treatment, and the urgency of achieving a protective effect. For example, a slow updosing protocol, with the maintenance dose only reached after several weeks to months, is of limited use if protection is to be achieved as quickly as possible to allow the patient to return to a hazardous exposure as soon as possible. For rapid updosing, a rush or ultra-rush protocol in a hospital is the method of choice. Conventional outpatient updosing is often preferred by patients and may be considered, especially if it can be done outside the insect season, and if the maintenance dose can be reached before the start of the next season. In the case of systemic side effects, the inpatient setting has advantages over the outpatient setting. 

**5.3.4. Maintenance therapy**

Once the maintenance dose has been reached, the intervals of the injections are gradually extended. The therapeutic allergens are then injected every 4 weeks for the 1^st^ year. Thereafter, depending on the preparation, the injection interval can be extended to every 5 – 6 weeks. If a depot preparation is used, the permissible injection interval can be 8 weeks from the 3^rd^ year (Box 16). 

### 5.4. Side effects of VIT 

**5.4.1. Local reactions**

Most patients experience significant redness and swelling at the injection sites during the dose-escalation phase of VIT. Symptoms diminish as treatment progresses, and can be treated symptomatically with a glucocorticoid cream and cold compresses. In addition, concomitant use of an H1-blocking antihistamine [127] or montelukast [128] (off label use), may suppress such reactions. Local reactions are reduced by the use of a depot preparation [116, 129] or highly purified aqueous therapeutic allergens [117]. 

**5.4.2. Systemic reactions**

Despite a history of sometimes life-threatening anaphylactic reactions, VIT is well tolerated by the vast majority of patients and significant side effects are rare. In the literature, systemic anaphylactic reactions and subjective common complaints such as fatigue, malaise, and headache associated with VIT are often collectively referred to as systemic adverse reactions, and the true incidence of VIT-induced SAR is often unclear. Overall, the incidence of systemic adverse reactions in the initial phase of treatment has been reported to range from 3.1% [130] to 50% [127], with severe reactions being very rare [53, 94, 102, 123, 125, 126, 127, 130]. Equipment, experienced staff, and knowledge of the management of a SAR are necessary prerequisites for the implementation of VIT [43]. 

Systemic adverse reactions are more common in the dose-escalation phase than in the maintenance phase [122], and are more common with bee venom than with vespid venom [53, 96, 102]. In patients with mastocytosis [131] or elevated bST [102], anaphylactic reactions are more common than in patients without evidence of mast cell disease. However, even in mast cell disease, systemic adverse reactions are usually mild; however, exceptionally severe reactions may occur in isolated cases [131]. Therefore, in patients with mast cell disease, it is recommended to perform updosing in an inpatient setting, if possible, and that maintenance therapy be managed with particular care. 

In addition, the incidence of anaphylactic reactions to VIT is significantly increased in patients on antihypertensive therapy [102], without attribution to any particular medication. This observation may reflect an increased risk of anaphylaxis in cardiovascular disease. In a prospective multicenter study investigating the effect of ACE inhibitors and β-blockers, neither the use of these drugs nor the presence of cardiovascular disease was associated with an increased risk of adverse reactions to VIT [53]. 

Concomitant use of an H1-blocking antihistamine was effective in preventing milder SAR [53, 127], but may not prevent more severe anaphylaxis [102, 127]. The efficacy of VIT is not affected by concomitant treatment with an H1-blocking antihistamine [132]. 

Anaphylactic reactions are treated symptomatically according to guidelines [43]. If an objective or significant subjective adverse reaction has occurred, the manufacturer’s instructions for use of the drug should be followed. Pragmatically, the procedure is individualized, depending on the severity of the SAR and the updosing regimen. In the case of mild reactions, an attempt may be made to continue the updosing regimen while being on antihistamine protection after the patient has recovered. For moderate reactions, updosing using a rush protocol can usually be continued after 8 – 12 hours of symptom relief. For continuation, the dose should be reduced by two categories and should then be increased again according to the protocol (Box 17). 

**5.4.3. Repeated anaphylactic reactions**

Repeated anaphylactic reactions during dose excalation or maintenance therapy are rare. If such reactions are severe, they may force discontinuation of therapy. As repeated SAR to VIT are predictors of treatment failure [59, 133], it is particularly important to establish tolerated therapy in these patients. 

An apparent paradox is that patients often tolerate a higher dose better than a lower dose, with the individual reaction threshold first having to be exceeded. AIT, on the other hand, requires the administration of a relatively high dose of allergen to achieve long-term tolerance. If a temporary dose reduction is required, the aim should be to increase the reduced dose back to the target dose as quickly as possible. In the event of recurrent systemic adverse events during maintenance or at initiation, a maintenance dose of 200 µg, or higher if necessary, is indicated. 

The recommended diagnostic and therapeutic approach for repeated SAR to VIT is summarized in Table 15; however, sustained tolerance to VIT can only be achieved in exceptional cases with the measures listed there in points 1 – 3. Severe anaphylactic reactions cannot be prevented by premedication with an H1-blocking antihistamine [102, 127]. 

If there are recurrent systemic reactions and risk factors, pre-treatment with an anti-IgE antibody (omalizumab, currently approved for treating urticaria, asthma, and nasal polyps) may be considered. As omalizumab is not approved for the prophylaxis of anaphylaxis, the modalities of off-label use need to be considered. One case series showed that tolerability of a previously intolerable VIT could be achieved by using omalizumab ~ 2 months prior to re-initiation and still overlapping 4 – 6 months after a higher maintenance dose had been reached [134]. In contrast, in several case reports, even a single administration of anti-IgE antibody was sufficient to ensure tolerance to VIT [135]. 

If pre-medication with an IgE antibody is not possible and a new updosing is not tolerated, it is recommended to continue with the highest tolerated HV dose permanently (Box 18). 

**5.4.4. Unusual side effects**

Unusual side effects of VIT are extremely rare and may include serum sickness-like reactions, granulomas at the injection site, or allergic vasculitis [97]. For all symptoms that occur in temporal connection with VIT, it must be checked whether they are causally related to the treatment. If this is the case, VIT should be continued on an individual basis. In the case of granulomas at the injection site, the use of an aluminium hydroxide-adsorbed preparation should be changed to an aluminium hydroxide-free preparation. 

### 5.5. Control of therapeutic efficacy, sting provocation 

During ongoing VIT, compliance is monitored, and factors that may require an increase in dose or prolongation of therapy are recorded. Monitoring includes:

- Medical history (especially tolerance of VIT, reaction to field stings, occurrence or course of comorbidities, use of medications),
- Verification of carrying, and durability of emergency kit medications, if applicable [136].

If VIT has been discontinued prematurely, it should be discussed whether re-initiation is indicated. 

It would be desirable to determine the clinical efficacy of VIT using laboratory parameters. VIT has a variety of immunological effects: at the beginning of treatment there is an increase in skin test reactivity to the therapeutic allergens and corresponding serum sIgE concentrations; later, skin test reactivity and insect venom sIgE concentration decrease and sometimes become completely negative. Concentrations of specific serum IgG antibody concentrations will also increase, but will remain elevated for longer periods [116, 137]. However, this does not allow verification of the onset of clinical protection. The only way to detect failure of VIT is to be stung by a live insect. 

For this purpose, a sting provocation can be carried out (see also section 3.5), the main indications of which are the detection of treatment failure and, in the more likely case, the improvement of quality of live by demonstrating to the patient that a sting is being tolerated. The latter has been demonstrated in several studies [92, 93, 95]. To find out whether further field stings will be tolerated, a tolerated sting provocation has a high predictive value; however, it does not provide proof of permanent protection [138]. 

To check the success of therapy, sting provocation under emergency preparedness should only be carried out in inpatients during ongoing, tolerated VIT, preferably 6 – 12 months after the start of therapy. In Germany and Austria, sting provocation is only offered by a few specialized centers. For reasons of resource allocation, sting provocation can be omitted in patients in whom therapeutic protection is likely to have occurred due to the overall constellation [59]. Conversely, if therapeutic failure is highly likely or already evident because a SAR to a field sting has occurred, sting provocation should only be performed after further updosing or therapy adjustment. Indicators of treatment failure include recurrent SAR to maintenance therapy. 

For a detailed description of the procedure, see a previous EAACI position paper [46]. If a SAR occurs during sting provocation, the therapeutic goal has not been achieved and therapy adjustment is indicated. The applied dose should then be increased by 50 – 100 µg of insect venom, which almost always results in complete protection [94, 139]. Tolerance achieved in this way can be tested with a new sting provocation. 

Children and adolescents should only undergo a sting challenge test in exceptional cases. Tolerance of field stings during VIT can be used to confirm the success of the therapy. However, a field sting tolerated without SAR is prognostically inferior to a tolerated sting provocation; possible reasons could be an irrelevant insect or insufficient venom delivery (sting in passing) [133] (Box 19). 

### 5.6. Therapeutic failure: risk factors and management 

In adults, the efficacy of VIT with bee venom is worse than with vespid venom [53, 59, 94, 96]. Similar observations have not been made in children [24, 63, 88]. The reasons for the poorer response to bee VIT in adults are unclear; a higher frequency of therapeutic failure may be due to the different composition of the venoms or a dose phenomenon, as a higher dose of venom is usually delivered by a bee sting compared to a *Vespula* sting. Other risk factors for treatment failure include mast cell disease (mastocytosis, elevated bST) and repeated SAR during VIT (Table 16). The use of ACE inhibitors was also found to be a risk factor for treatment failure in a retrospective study [59], although no evidence was found in a prospective study [53]. If there is evidence of treatment failure during ACE inhibitor medication, discontinuation of the drug should be considered. 

In the case of treatment failure, i.e., the occurrence of a SAR to a new sting, an increase in the maintenance dose can almost always still achieve therapeutic success [94, 139]. In most cases, 200 µg is sufficient, occasionally 300 µg and up to 400 µg may be required, especially in patients with bee venom allergy, but rarely in vespid venom allergy. There is no empirical evidence for using higher doses. The establishment of protection after increasing the dose should again be checked by sting provocation. 

Very rarely, an increase in the maintenance dose does not provide adequate protection against sting reactions. For these patients, prophylactic treatment with an anti-IgE antibody during the stinging season is recommended. In the absence of drug approval for this indication, the modalities of off-label use apply (Box 20). 

### 5.7. Therapy duration 

Maintenance therapy should be given every 4 – 6 weeks (up to every 8 weeks for depot preparations) for at least 3 and usually 5 years in adults, and 3 – 5 years in children. In children and adolescents who initially had a mild to moderate systemic reaction (grade I or II), VIT may be discontinued after 3 years.

- The decision to discontinue or continue therapy for extended periods of time should be based on individual characteristics of the patient:
- Recurrent adverse reactions while being on VIT maintenance therapy
- SAR to a sting by the culprit insect while being on maintenance therapy, and lack of confirmation of the efficacy of VIT when using an increased maintenance dose.
- In individual cases, VIT may be prolonged for quality of life reasons (patient request).
- If there is a particular insect exposure, VIT is given beyond the usual treatment period until the end of intensive contact (e.g., when beekeepers stop beekeeping). In this case, VIT should be continued for 6 months after the last sting.
- Long-term VIT may be considered in patients with

   – mastocytosis (possibly also in cases only presenting with an increased bST > 20 µg/L in the absence of proven mastocytosis) 

   – cardiovascular or respiratory arrest due to Hymenoptera sting anaphylaxis 

   – presence of other, exceptionally strong risk factors (e.g., hereditary α-tryptas emia). 

Even after discontinuation of VIT, the protective effect persists in many patients but is lost in up to 20% of patients within 5 –  10 years [140, 141]. This observation indicates that sustained protection can only be expected with continued therapy. 

The longer duration of therapy, the greater the efficacy of VIT [59]. For long-term VIT, extending the injection interval would facilitate treatment. If the extended injection interval is not consistent with the product information of the manufacturer, off-label use modalities must be considered. In some patients, SAR due to VIT injections will occur when extending the injection interval [142, 143]. For VIT with bee venom, it has been shown that the percentage of patients losing protection (25%) will be unacceptably high, if injections are given only every 6 months [142]. According to other studies, however, such extended time intervals may be safe as well for VIT with bee and vespid venom [143, 144] (Box 21). 

### 5.8. Follow-up after completion of Hymenoptera venom specific immunotherapy 

Even after VIT has been completed, measures are required to prevent recurrent stings (Table 8) and, if necessary, to ascertain that patients carry the personal emergency kit, if applicable (Table 9). If permanently carrying an emergency kit is indispensable, the patient should see an allergist once a year so that the durability of the medication can be checked and the dose adjusted if necessary; training on how to use the kit is also required. In the event of a new SAR to a Hymenoptera sting, the patient should consult a doctor immediately. Allergy work-up and, if necessary, re-initiation of VIT is then required. 

## Currently unresolved questions and problems

- The manufacturers of therapeutic allergens should harmonize the contraindications listed and adapt them to the current state of knowledge.
- Prospective, randomized studies with a sufficient number of cases are needed to answer the following questions:

   – What is the duration and dose of anti-IgE antibody as adjunctive therapy in patients who poorly tolerate VIT? 

   – What is the tolerability and efficacy of different bee venom preparations depending on individual allergen recognition patterns?

- There is a lack of systematically collected data on tolerability and efficacy when extending therapeutic intervals, or switching between different commercially available products.

## Funding 

The digital conferences (GoToTraining) and the moderator were financed by the DGAKI. In addition, no compensation was paid. 

## Conflict of interest 

The conflicts of interest were recorded using the AWMF portal interessenerklaerungonline.de, evaluated by the conflict of interest officer of the DGAKI (for details see the guideline report) and tabulated in accordance with the AWMF. The guideline report and conflict of interest table are available at www.awmf.org/leitlinien/. 

**Abbreviations Abbreviations:** Abbreviations.

AAI	Adrenaline auto-injector
ABD	Working Group for Occupational and Environmental Dermatology e.V.
AeDA	Medical Association of German Allergologists
AIT	Allergen immunotherapy
AWMF	Association of the Scientific Medical Societies
bST	Baseline serum tryptase concentration
CCD	Cross-reactive carbohydrate determinants
DDG	German Society of Dermatology
DELBI	German Guideline Assessment Tool
DGAKI	German Society for Allergology and Clinical Immunology
DGHNO-KHC	German Society of Oto-Rhino-Laryngology, Head and Neck Surgery
DGKJ	German Society of Pediatrics and Adolescent Medicine
DGP	German Respiratory Society
EAACI	European Academy of Allergology and Clinical Immunology
GPA	Society for Pediatric Allergy and Environmental Medicine
H1	Histamine 1
HB	Honey bee
HIV	Human immunodeficiency virus
HV	Hymenoptera venom
HVA	Hymenoptera venom allergy
HVS	Hymenoptera venom sensitization
HV-sIgE	Hymenoptera venom-specific IgE antibodies
IL	Interleukin
ÖGAI	Austrian Society for Allergy and Immunology
SAR	Systemic allergic reactions
sIgE	Specific IgE antibodies
VIT	Venom immunotherapy
VV	Vespid venom

**Table 1.** Participating organizations and delegated representatives.

German Respiratory Society (DGP)	Dr. Wolfgang Sieber Norbert K. Mülleneisen
German Society for Allergology and Clinical Immunology (DGAKI)	Prof. Dr. Margitta Worm Prof. Dr. Knut Brockow Univ.-Prof. Dr. Thilo Jakob Prof. Dr. Bettina Wedi Prof. Dr. Franziska Ruëff
German Society of Dermatology (DDG)	Prof. Dr. Ulf Darsow Prof. Dr. Regina Treudler Prof. Dr. Wolfgang Pfützner Dr. Jörg Fischer
Austrian Society for Allergy and Immunology (ÖGAI)	Prof. Dr. Wolfram Hötzenecker Priv.-Doz. Mag. Dr. Stefan Wöhrl
Medical Association of German Allergists (AeDA)	Prof. Dr. Randolf Brehler Prof. Dr. Thomas Fuchs Univ.-Prof. Dr. Hans Merk Prof. Dr. Ludger Klimek
German Society of Oto-Rhino-Laryngology, Head and Neck Surgery (DGHNO-KHC)	Priv.-Doz. Dr. Adam Chaker Priv.-Doz. Dr. Sven Becker
Society for Pediatric Allergy and Environmental Medicine (GPA)	Dr. Sunhild Gernert Dr. Michael Gerstlauer Dr. Irena Neustädter
Arbeitsgemeinschaft für Berufs- und Umweltdermatologie e.V. (Association for Occupational and Environmental Dermatology, ABD)	Prof. Dr. Andrea Bauer Prof. Dr. Christoph Skudlik
German Society for Pediatrics and Adolescent Medicine (DGKJ)	Dr. Lars Lange Prof. Dr. Eckhard Hamelmann

**Table 2.** Recommendation strengths.

**Strength**	**Syntax**
Strong recommendation	Shall
Weak recommendation	Should
Open recommendation	Can

**Table 3.** Severity scale for the classification of anaphylactic reactions (according to Ring and Messmer) [7]*.

**Grade**	**Skin#**	**Abdomen**	**Respiratory tract**	**Cardiovascular system**
I	Itch Flush Urticaria Angioedema	–	–	–
II	Itch Flush Urticaria Angioedema	Nausea Cramps	Rhinorrhea Hoarseness Dyspnea	Tachycardia (increase of heart rate ≥ 20/minutes) Hypotension (decrease of systolic blood pressure ≥ 20 mmHg) Arrhythmia
III	Itch Flush Urticaria Angioedema	Vomiting Defecation	Laryngeal Edema Bronchospasm Cyanosis	Shock Loss of consciousness
IV	Itch Flush Urticaria Angioedema	Vomiting Defecation	Respiratory arrest	Cardiac arrest

^#^Generalized skin symptoms apart from the sting area; *Classification is based on the most severe symptoms encountered (none of the symptoms is obligatory).

**Box 1.** Recommendations on the indication of allergological testing (skin test, IgE detection).

	Strength of consensus
1. If there is a history of a general allergic reaction after a Hymenoptera sting, allergy testing shall be performed.	Strong
2. Without evidence of a general allergic reaction after Hymenoptera sting(s) („exclusion of insect venom allergy“), no diagnostic procedures should be undertaken.	Majority
3. If therapeutic consequences are unlikely because of only a mild systemic reaction limited to the skin, allergy testing should be avoided.	Majority

**Table 4.** Questionnaire for taking medical history in case of a systemic insect sting reaction.

Insect venom allergy questionnaire								
Date					Patient: female □ male □			
Weight: kg		Height: cm			Severity of reaction			
		1^st^ sting	2^nd^ sting	3^rd^ sting	Symptoms	1^st^ sting	2^nd^ sting	3^rd^ sting
Sting date (day/month/year)					Itching all over the body			
Insect	Bee				Heat sensation			
	*Vespula*				Rash all over the body			
	Other				Tingling in hands/feet			
	Certain				Face swelling			
	Uncertain				Runny nose			
Localization of the sting					Redness of the eye conjunctiva			
Interval until symptom onset (min/h)					Lump/tightness in the throat			
Site and circumstances of the event					Cough irritation			
Physical effort?					Shortness of breath			
Mental stress when reacting?					Nausea			
Did the sting remain in the skin?					Vomiting			
Occupation?					Urinary (stool) urgency/discharge			
Outdoor activities?					Dizziness			
Later tolerated stings? Yes □ No □					Feeling of weakness (circulatory disorder)			
Beekeeper? Yes □ No □					Headache			
Is there a beekeeper in the neighborhood? Yes □ No □					Unconsciousness (duration)			
Other					Other			
Hay fever □ Asthma □ Atopic eczema □					Treatment: self/doctor			
Comorbidities					Adrenaline			
					Glucocorticoid			
					Antihistamines			
					Intravenous fluids			
					Hospital admission			
Medication at reaction (R) or currently (C)					Intensive Care Unit			
					Recovery after (hour(s)/day(s)/week(s))			
					Information sheet handed out Yes □ No □			
					Emergency kit available Yes □ No □			
					Adrenaline auto-injector (trade name)			
					Other medications:			

**Table 5.** Clues about the kind of insect causing the reaction [44].

**Bee**	**Vespid**
Rather “peaceful” (except at the hive)	Rather “aggressive”, sting can also occur in “passing flight”.
Main flying season spring to late summer(even on warm winter days!)	Main flying season summer until late autumn
After a sting, the stinger usually remains in the skin	Sting usually does not remain in the skin (exceptions are possible due to shearing, if the insect was trapped, for example)
Occurrence mainly in the vicinity of bee hives, flowers, and clover	Occurrence mainly in the vicinity of food or garbage

**Table 6.** Variables increasing exposure risk.

(Hobby) beekeepers, family members and neighbors of beekeepers
Professions such as fruit or bakery seller, forestry worker, gardener, firefighter, farmer, roofer, construction worker
Intensive practice of outdoor activities

**Box 2.** Recommendations on the recording of risk factors.

	**Strength of consensus**
4. Risk factors for an increased sting risk shall be obtained when taking the medical history.	Strong
5. The medical history shall capture possible risk factors for more severe anaphylaxis.	Strong
6. If a systemic allergic reaction has not only affected the skin, basic diagnosis for the detection of mastocytosis shall involve a skin inspection to detect mastocytosis of the skin and a determination of basal serum tryptase concentration.	Consensus

**Box 3.** Recommendations on the in-vitro diagnostics of sIgE against Hymenoptera venoms and their components.

	**Strength of consensus**
7. A determination of specific IgE antibodies against bee and/or *Vespula* venom/components shall be performed; in case of a suspected sting reaction caused by other Hymenoptera, this determination shall be also directed against the corresponding other venom.	Strong
8. In the case of negative test results obtained shortly (less than 2 weeks) after the sting reaction, the tests shall be repeated (no sooner than 4 – 6 weeks after the sting reaction).	Strong
9. In case of double sensitization against whole bee and *Vespula* venom extract, or if an implausible result is suspected, testing of sIgE against recombinant components shall be performed.	Strong

**Box 5.** Recommendations on the determination of bST.

	**Strength of consensus**
12. All patients with anaphylaxis (severity grade ≥ II) after a Hymenoptera sting shall have a determination of bST.	Strong
13. In case of elevated serum tryptase measured within 24 hours after the acute sting event, a control measurement shall be performed in the symptom-free interval.	Strong
14. If the bST concentration is permanently elevated (> 20 µg/L), further diagnostic measures shall be performed to clarify mastocytosis.	Consensus

**Box 6.** Recommendations on skin tests with Hymenoptera venoms.

	**Strength of consensus**
15. If an unequivocal diagnosis is obtained by in-vitro diagnostics, a skin test can be omitted.	Consensus
16. The skin test can be performed as a titrated prick and/or intradermal test.	Strong

**Box 7.** Recommendation on sting provocation in adults.

	**Strength of consensus**
17. Diagnostic sting provocations (before the start of VIT) or sting provocations after completion of VIT shall not be performed.	Consensus

**Box 8.** Recommendations on large local sting reactions.

	**Strength of consensus**
18. Acute treatment can be symptomatic using non-sedating antihistamines, cooling compresses, topical and/or systemic glucocorticoids.	Consensus
19. Antibiotic therapy for the treatment of non-infectious lymphangitis or lymphadenopathy shall not be performed.	Consensus

**Box 9.** Recommendations for long-term care in patients with a history of a large local reaction.

	**Strength of consensus**
20. VIT shall not be performed for large local reactions.	Strong

**Table 9.** Emergency medication for self-treatment in children and adults [43].

	Adrenaline auto-injector for intramuscular application, weight-adapted:
7.5 – 25 kg BW or 15 – 30 kg BW	150 μg*
25 – 50 kg BW or 30 – 50 kg BW	300 μg*
> 50 kg BW	300 – 500^#^ – 600^#^ μg
– H1 receptor-blocking antihistamine, according to patient age and preference, orally as liquid or (melting) tablet – The dose of the respective antihistamine can be increased off-label up to four times the single dose – For dimetinden drops, a weight-adapted dosage of the IV formulation can be recommended as an oral dose (Table 8)	
Glucocorticoid, according to patient age and preference, rectally or orally (as liquid or tablet) with 50 – 100 mg prednisolone equivalent	
In case of known bronchial asthma or previous reaction with bronchospasm additionally β2-adrenoceptor agonist 2 puffs	
If there is a history of laryngeal edema, additionally: inhaled adrenaline preparation with spray head for drug vial (to be specifically requested from pharmacist)	

Note: An emergency first aid kit should include an anaphylaxis passport with written instructions for use of the components. *According to the respective approval status for the prescribed autoinjector; BW = body weight; ^#^not available in Austria; IV = intravenous.

**Table 11.** Recommendations for prescribing AAIs in patients with insect venom allergy.

**Absolute indication**
– Children and adults with mastocytosis and/or elevated basal serum tryptase levels: before, during, and after completion of immunotherapy
– Untreated children and adults with more than cutaneous/mucosal SAR (i.e., grade I anaphylaxis) and at high risk of re-exposure
– During VIT: in children and adults with more than cutaneous/mucosal SARs (i.e., grade I anaphylaxis) when there are additional risk factors* for non-response to immunotherapy
– After completion of regular VIT in children and adults presenting with more than cutaneous/mucosal SAR (i.e., grade I anaphylaxis) and if there are additional risk factors* for non-response to VIT.
**Relative indication**
– Long distance to medical care and/or high risk of exposure and/or impaired quality of life
– After completion of regular VIT in children and adults with cutaneous/mucosal reactions (grade I) who are at increased risk of exposure and/or have had a short duration of immunotherapy (< 3 years)
– Individual patient request

*Risk factors in this context are severe insect venom anaphylaxis (grade III or IV), high risk of exposure (e.g., beekeeper), (repeated) systemic reaction under immunotherapy, mastocytosis, or elevated baseline serum tryptase above 20 µg/L . For adults, bee venom allergy is also considered a risk factor.

**Table 10.** Patient information sheet “How to behave in the event of a sting”.

– Keep calm! If attacked by bees or wasps, protect the head with arms or clothing. The retreat must not be hectic, but very slow. Insects release pheromones when stinging, which also motivate other insects to sting. Therefore, the sting site should be covered with the hand in the event of a sting.
– Try to selectively inform bystanders about the sting event and its possible consequences.
– Immediately remove any stinger remaining in the skin. When doing so, do not squeeze the sting apparatus with your fingers, but scrape it away to the side.
Emergency medication in case of mild reactions limited to the skin:
– If venom-specific immunotherapy has not yet been administered, oral medication is taken immediately after the sting, even in the absence of symptoms, according to the doctor‘s instructions:
– Antihistamines
– Steroids
– After a successful allergen-specific immunotherapy*, medication should only be taken if, contrary to expectations, systemic symptoms do occur. For symptoms limited to the skin, oral medications are used first, and for more extensive reactions, the adrenaline auto-injector is used.
Emergency measures in case of shortness of breath, swelling in the mouth/throat region or of circulatory problems:
– Inject adrenaline laterally into the lateral thigh
– In case of asthma, inhale 2 puffs of the emergency spray
– Correct positioning (shortness of breath→ raised upper body, circulatory problems head-down position, unconsciousness→ stable side position)
– Take oral medications only if swallowing is possible without problems
– Alert an emergency doctor immediately!

*Your allergist has confirmed that success of an allergen-specific immunotherapy is highly likely based on a tolerated sting provocation or field sting.

**Box 10.** Recommendations on the emergency kit.

	**Strength of consensus**
21. In patients with a history of a severity grade I reaction, and in the absence of other risk factors, the prescription of an AAI is not required. However, the AAI can be prescribed in special situations (e.g., high risk of exposure, long distance to medical care, limitation of quality of life).	Consensus
22. In patients with a history of anaphylaxis (grade II – IV) or of a severity grade I reaction in combination with a high risk of re-exposure, an emergency kit including an AAI shall be prescribed until allergy diagnosis and assessment are complete.	Consensus
23. After successful initiation of VIT and reaching the maintenance dose at the maintenance interval, the prescription of an AAI can be waived in patients with a history of a systemic sting reaction (severity grade I – II) and in the absence of other risk factors for VIT failure.	Consensus
24. After successful completion of VIT, the prescription of an AAI can be waived in patients with a history of a systemic sting reaction (severity grade I – II) and in the absence of other risk factors for VIT failure.	Consensus
25. Patients with grade III or IV anaphylaxis or patients who present with other risk factors for VIT failure shall carry an emergency kit with an AAI during and after VIT. Risk factors include: high risk of exposure (e.g., beekeepers), repeated SAR on immunotherapy, mast cell disease, and/or elevated basal serum tryptase (> 20 µg/L). For adults, bee venom allergy is also considered a risk factor.	Consensus

**Box 11.** Recommendation on ACE inhibitors.

**Recommendation on ACE inhibitors**	**Strength of consensus**
26. If there is no firm need for the use of ACE inhibitors and if their switching is straightforward, the drug may be replaced by another medication.	Consensus

**Box 12.** Recommendations on the indication of Hymenoptera VIT.

	**Strength of consensus**
27. VIT shall be performed in patients with a history of an anaphylactic reaction of severity grade ≥ II according to Ring and Messmer, and with evidence of IgE-mediated sensitization to the culprit venom.	Strong
28. If there is increased exposure, if there are relevant risk factors for a particularly severe anaphylaxis, and/or if quality of life would be significantly impaired without VIT, VIT shall be performed even if there is only a history of an exclusively cutaneous SAR.	Strong

**Table 12.** Contraindications of VIT.

Uncontrolled asthma
Active malignant neoplastic diseases
Severe active systemic autoimmune diseases and severe immunodeficiencies
Insufficient compliance
Untreated, chronic infection (e.g., active HIV, viral hepatitis)

**Box 19.** Recommendation on sting provocation.

	**Strength of consensus**
43. Sting provocation can be performed on a case-by-case basis to verify the success of therapy. Provocation shall only be performed in patients who have reached the planned maintenance dose and tolerate VIT.	Strong

**Table 16.** Variables associated with treatment failure/success [59].

Risk factors or predictors of treatment failure
– Bee venom > *Vespula* venom
– Repeated systemic allergic reactions while being on VIT
– Mastocytosis, increased bST
Protective factors
– Higher treatment dose (also double VIT)
– Extended treatment time

**Box 20.** Recommendations on the management of systemic allergic sting reactions while being on maintenance therapy.

	**Strength of consensus**
44. If treatment failure is evident during ACE inhibitor therapy, discontinuation of the ACE inhibitor should be considered.	Strong
45. If there is evidence of overt therapeutic failure, maintenance venom dose shall be increased in adults to up to 200 µg or above (maximum 400 µg), and in children to up to 200 µg.	Strong
46. If protection cannot be established by increasing the maintenance dose and if there are co-factors for severe anaphylaxis, co-medication with an IgE antibody (omalizumab; off-label use) should be considered during the relevant insect flight period.	Strong

**Table 7.** Allergologically significant components of bee and Vespula venom (http://www.allergome.org).

**Apis mellifera**		**Vespula species**	
Api m 1	Phospholipase A2^a#^	Ves v 1	Phospholipase A1^a#^
Api m 2	Hyaluronidase^a,b#^	Ves v 2	Hyaluronidase^a,b^
Api m 3	Acid phosphatase^a#^	Ves v 3	Dipeptidyl peptidase^a,b#^
Api m 4	Melittin^c#^	Ves v 5	Antigen 5^a^
Api m 5	Dipeptidyl peptidase^a,b#^	Ves v 6	Vitellogenin^b^
Api m 6	Protease inhibitor		
Api m 7	CUB Serine Protease		
Api m 8	Carboxylesterase		
Api m 9	Serine carboxypeptidase		
Api m 10	Icarapine^a#^		
Api m 11	Gellée royal protein		
Api m 12	Vitellogenin^b^		

^a^Major allergen: More than 50% of the patients tested show sensitization to the allergen in question; ^b^cross-reacting venom allergens. The sIgE reactivity against bee venom hyaluronidase can be interpreted as a marker for bee venom-specific sensitization. In contrast, sIgE reactivity against *Vespula* venom hyaluronidase is mainly based on reactivity against cross-reactive carbohydrate determinants; ^c^research purposes; ^#^IgE detection kits for single detection are commercially available (singleplex).

**Table 8.** Measures to prevent Hymenoptera stings.

– Repellents (chemical insect repellents) do not provide protection.
– When being outdoors, avoid eating or drinking food, picking fruits or flowers, staying near waste baskets, trash cans, animal enclosures, or fallen fruit, and using perfume or scented cosmetics. Wash hands and wipe mouth after eating.
– Do not drink from bottles or beverage cans, cover drinking glasses, use straws.
– Do not scare insects away from food sources, especially not with hectic movements.
– Keep skin largely covered by clothing (at least when gardening). Do not walk barefoot, or use open foot wear. When riding a motorcycle, wear gloves and motorcycle clothing close to the skin. Open bicycle helmets are to be provided with a net.
– Be especially careful on days with hot and humid weather, as insects are aggressive during such weather.
– Avoid wearing loose-fitting, light garments, e.g., loose skirts or dresses with dark colors; try to wear dresses with light colors.
– Keep apartment windows closed during the day or secure them with insect nets. No light in the evening when windows are open, as hornets are nocturnal and then prefer to fly towards light sources.
– Watch for hidden insects (especially in bed or shoes).
– Beehives must be avoided. Nests near a permanent residence must be removed (by beekeeper or fire department).
– Wasp traps or repellent sprays can be helpful.
– When approached by insects or being near the nest, avoid hectic or flapping movements, pull back slowly! Nests must not be shaken. Do not breathe into a flight hole.

**Box 4.** Recommendations on IgE determination against Hymenoptera venoms and their components.

	Strength of consensus
10. If HVA requiring absolutely necessary treatment is suspected, and if results from IgE detection methods for venom components and whole venom and from skin tests are not conclusive, cellular tests can be performed.	Consensus
11. Determination of specific IgG antibodies to Hymenoptera venom should not be used to assess the need for treatment of HVA.	Consensus

**Table 13.** Schemes* for updosing to 100 µg insect venom.

**Period**		**Hymenoptera venom dose in µg**		
Day	Hour	Ultra-rush	Rush (3 days)	Cluster
1	0	0.01	0.02	0.02
	0.5	0.1	0.04	
	1	1	0.08	0.04
	1.5	10	0.2	
	2	20	0.4	0.08
	2.5	40	0.8	
	3	80	2	
	3.5		4	
	4.0		8	
2	0	100	8	
	2		20	
	4	100	40	
	6		80	
3	0		80	
	2		100	
Week	Hour			
1	0			0.2
	1			0.4
2	0			0.8
	1			2
4	0			4
	1			8
5				10
6				20
7				40
8				80
9				100

*There are numerous modifications to these updosing schemes in which the maintenance dose of 100 µg can be reached in a shorter or longer time, and which contain even more or fewer intermediate steps.

**Table 14.** Updosing schemes with aqueous Hymenoptera venom [according to Ruëff (scheme 1) or Bauer (scheme 2)] to > 100 µg).

		**Scheme 1**	**Scheme 2**
**Day**	**Minutes**	**Dose (µg)**	**Dose (µg)**
1	0	100	100
	+30	20	40
	+30	30	60
2	0	150	100
	+30	20	100
	+30	30	
3		200	

**Table 15.** Management of repeated systemic allergic reactions to Hymenoptera VIT.

1. Identification (and, where possible, elimination) of risk factors for SARs in VIT.
Drugs Concomitant inhalant or food allergy Chronic infection, other general diseases Physical exertion on the day of injection
Optimization of drug therapy at the reacting organ (for example, an anti-obstructive therapy for asthmatic reactions).
2. Adjunctive therapy with H1-blocking antihistamine
3. Continued administration of the highest tolerated dose of HV for 3 months, then starting updosing again
4. Pretreatment with an anti-IgE antibody (300 mg omalizumab; off-label use): e.g., 5, 3, and 1 week before resuming updosing (> 100 µg maintenance dose if necessary) and subsequent continuation every 4 weeks for 4 – 6 months [134].

SAR = systemic allergic reactions; VIT = venom immunotherapy; HV = hymenoptera venom

**Box 13.** Recommendations on contraindications of Hymenoptera VIT.

	**Strength of consensus**
29. The use of β-blockers and ACE inhibitors are no contraindication of VIT. Patients should be informed about possible interactions.	Consensus
30. The following contraindications to VIT shall be respected: uncontrolled bronchial asthma, active malignant neoplastic disease, severe active systemic autoimmune disease and severe immunodeficiency (terminal AIDS), inadequate compliance, untreated chronic infection (e.g., active HIV, hepatitis C), pregnancy (for re-initiation)	Strong
31. In individual cases, VIT may be applied despite the presence of contraindications. This concept shall include a thorough weighing of the benefits and risks. Autoimmune diseases, severe cardiovascular or pulmonary diseases, and malignant diseases shall be optimally treated, shall be in remission before initiation of VIT, and shall be closely monitored during the course of VIT.	Strong

**Box 15.** Recommendations on venom selection for Hymenoptera VIT.

	**Strength of consensus**
34. For VIT, that venom shall be used, which was the culprit venom according patient history and to the results of the allergological work-up.	Strong
35. If there is double sensitization, if the history of the patient is uncertain with regard to the culprit venom, and if the culprit venom cannot be determined even by additional diagnostic procedures, VIT with both venoms shall be performed.	Strong
36. If allergy to the venoms of bumblebees or hornets is certain, VIT shall be performed with the related, partly cross-reacting venoms of bees or wasps.	Consensus

**Box 14.** Recommendations on the practical implementation of Hymenoptera VIT.

	**Strength of consensus**
32. The standard maintenance dose of VIT shall be 100 µg of Hymenoptera venom.	Strong
33. In case of bee venom allergy and increased risk of sting or risk of particularly severe anaphylaxis, starting VIT with a maintenance dose of 200 µg may be considered.	Consensus

**Box 16.** Recommendation on the maintenance therapy of Hymenoptera VIT.

	**Strength of consensus**
37. The maintenance dose should be administered at 4-week intervals in the 1^st^ year and, taking into account the manufacturer’s information, can be administered every 5 – 6 weeks from the 2^nd^ year onwards, depending on the preparation used, and every 8 weeks from the 3^rd^ year onwards if a depot preparation is used.	Consensus

**Box 17.** Recommendation on the reduction of side effects in Hymenoptera VIT.

	**Strength of consensus**
38. A non-sedating antihistamine can be administered as a preventive measure during updosing, which can be continued in the further treatment if required. In case of reactions in the area of the injection site, local cooling measures shall be used.	Consensus

**Box 18.** Recommendations on the management of repeated systemic allergic adverse events in Hymenoptera VIT.

	**Strength of consensus**
39. Possible risk factors of systemic side effects of VIT shall be identified and eliminated as appropriate.	Majority
40. Concomitant therapy with an H1-blocking antihistamine should be performed. The last tolerated dose should be continued for 3 months and, subsequently, a new updosing should be attempted.	Consensus
41. If risk factors for systemic side effects are present and cannot be eliminated, and if concomitant therapy with an H1-blocking antihistamine is not effective, concomitant treatment with an anti-IgE antibody (omalizumab; off-label use) should be performed.	Majority
42. If side effects continue to occur, the last maximum dose that was tolerated should be administered every 4 weeks for 5 years.	Consensus

**Box 21.** Recommendations on the duration of Hymenoptera VIT.

	**Strength of consensus**
47. In the absence of risk factors described below (recommendations 48 and 49), VIT can be discontinued after 3 – 5 years, provided that maintenance therapy has been tolerated without recurrent anaphylactic side effects.	Consensus
48. Permanent VIT can be considered in patients with, among others, – established mastocytosis, – cardiovascular or respiratory arrest due to Hymenoptera stings – other specific individual constellations indicating an increased individual risk (e.g., hereditary α-tryptasemia)	Strong
49. If insect exposure time is greatly increased and unavoidable (e.g., occupational), VIT can be given to adults until the end of intensive contact.	Consensus
